# A CSF NPTX2-based residual metric captures prognostic heterogeneity within a proposed compensatory framework in prodromal Alzheimer’s disease

**DOI:** 10.1186/s13195-026-02160-y

**Published:** 2026-08-11

**Authors:** Federico Massa, Anastasia Lechiara, Francesca De Cesari, Virginia Pelagotti, Alessia Panza, Giulia Bozzo, Martina Urbinati, Mattia Losa, Stefano Raffa, Emanuela Maria Mobilia, Federica Maria Bozzano, Davide Visigalli, Luigi Lorenzini, Andrea Brugnolo, Nicola Girtler, Dario Arnaldi, Angelo Schenone, Antonio Uccelli, Gianmario Sambuceti, Silvia Morbelli, Giampaola Pesce, Matteo Pardini

**Affiliations:** 1 Department of Neuroscience, Rehabilitation, Ophthalmology, Genetics, Maternal and Child Health (DINOGMI), University of Genoa, Largo Daneo 3, Genoa, 16132 Italy; 2 AOM - IRCCS Ospedale Policlinico San Martino, Genoa, Italy; 3 Autoimmunology Laboratory, AOM - IRCCS Ospedale Policlinico San Martino, Genoa, Italy; 4 Department of Neuroscience Rita Levi-Montalcini, University of Turin, Turin, Italy; 5 Department of Radiology and Nuclear Medicine, Amsterdam UMC, Amsterdam Neuroscience, Vrije Universiteit, Amsterdam, Netherlands; 6 Department of Health Science (DISSAL), University of Genoa, Genoa, Italy; 7 Department of Medical Sciences, University of Turin, Turin, Italy; 8 Nuclear Medicine Unit, Azienda Ospedaliero-Universitaria Città della Salute e della Scienza di Torino, Turin, Italy

**Keywords:** Cerebrospinal fluid biomarkers, [^18^F]FDG PET, Synaptic dysfunction, Mild cognitive impairment (MCI), Alzheimer's disease, Neuronal pentraxin-2, Neurogranin

## Abstract

**Background:**

In Alzheimer’s disease(AD), core CSF biomarkers incompletely predict clinical progression. Synaptic biomarkers may add prognostic information by capturing distinct biological dimensions, from synaptic injury to adaptive network function. Although correlated, neurogranin (Ng) is interpreted as a marker of post-synaptic injury, whereas neuronal pentraxin-2(NPTX2) is more closely related to circuit homeostasis. We tested whether CSF NPTX2 contains a specific component statistically associated with a putative adaptive network signal, interpreted within a hypothesized compensatory framework, using clinical progression and brain metabolism as convergent readouts.

**Methods:**

We retrospectively studied 104 patients with typical MCI-AD. CSF core AD biomarkers, NPTX2 and Ng were measured with commercial immunoassays. Early and late MCI stages were defined by proximity to dementia conversion (LMCI ≤ 2 years; EMCI > 2 years or stable). A residual NPTX2 measure(Res_NPTX2) was derived by regressing z-transformed NPTX2 on Ng, an index of AD burden based on tau/amyloid ratio, and age. Associations with MMSE and time-to-conversion were tested using linear and survival models. In all patients, brain [^18^F]FDG PET was analyzed with voxel-based methods to identify metabolic correlates of NPTX2 and Res_NPTX2 relative to AD-related hypometabolic regions.

**Results:**

Ng correlated strongly with NPTX2 (ρ = 0.67,*p* < 0.001). Res_NPTX2 was higher in EMCI than LMCI (*p* = 0.0006), associated with better baseline MMSE (*p* = 0.008), and predicted less MMSE decline over time (*p* = 0.002). During follow-up, 55/104 patients(52.9%) progressed to dementia. Higher Res_NPTX2 predicted lower progression risk (HR = 0.63,*p* = 0.007), including after accounting for baseline MMSE. Kaplan–Meier curves differed across Res_NPTX2 tertiles, with the strongest separation within 2 years (high vs. low: HR = 0.18,*p* = 0.008). On [^18^F]FDG PET, LMCI showed more marked left-sided posterior hypometabolism than EMCI. CSF NPTX2 and Res_NPTX2 were associated with distinct metabolic patterns centered on prominently left posterior parietal and right fronto-striatal regions, respectively.

**Conclusions:**

In prodromal AD, CSF NPTX2 contains clinically relevant information beyond Ng and core biomarker burden. The proposed residualization approach provides a statistical strategy to isolate an NPTX2-related component associated with better cognition, lower short-term progression risk, and a precise right fronto-striatal metabolic signature. Within a hypothesized compensatory framework, this statistically isolated component may index a putative adaptive network capacity and help characterize clinically meaningful heterogeneity in early AD.

**Supplementary Information:**

The online version contains supplementary material available at https://doi.org/10.1186/s13195-026-02160-y.

## Introduction

Alzheimer’s disease (AD) is increasingly defined through in vivo biomarkers, with amyloid-beta and tau providing a biologically grounded basis for diagnosis and staging of AD pathology across the clinical spectrum, from the prodromal stage, i.e., mild cognitive impairment (MCI), to dementia [1, 2]. However, even within biomarker-confirmed MCI-AD, clinical trajectories diverge substantially. Some patients progress to dementia rapidly, whereas others remain stable or progress slowly over several years, despite broadly similar amyloid-tau profiles [3]. This variability impacts counseling, trial enrichment, and the choice and timing of disease-modifying interventions and is possibly explained by tau molecular diversity and spreading, co-pathology, as well as inter-individual differences in coping with AD pathology [2, 4–6]. Reserve and resilience frameworks emphasize that individuals differ in network properties and ability to recruit alternative pathways, so that comparable pathological burden may correspond with different cognitive outcomes across subjects [7–9]. Clinical progression can therefore be framed as a transition from network coping to failure. In early stages, networks may compensate for accumulating pathology through hyper-signaling and -connectivity, maintaining cognitive performance despite biological stress; over time, these mechanisms may become insufficient, leading to accelerated decline across multiple domains and progression to dementia [7, 10].

If clinical trajectories are shaped by downstream circuit integrity and adaptive capacity, then core markers alone may be insufficient to explain heterogeneity within biomarker-confirmed MCI-AD [2]. This gap has fueled interest in biomarkers that are closer to the biological substrate of cognition. Synaptic dysfunction is widely viewed as an early process in AD [11], correlating strongly with cognitive impairment and supporting the notion of AD as a “synaptic failure” model at the clinical level [12]. Accordingly, cerebrospinal fluid (CSF) synaptic proteins have emerged as candidate markers of downstream network status that may provide complementary and additive information beyond amyloid and tau [13, 14].

Importantly, synaptic biomarkers are not a single interchangeable class. Different proteins map onto distinct synaptic compartments (pre- vs. post-synaptic) and biological processes and therefore may index different axes of synaptopathy [13]. CSF Neurogranin (Ng) is among the best-established biomarkers and is typically interpreted as a dendritic marker of post-synaptic injury, with relatively specific increases in AD [15, 16]. In large-scale proteomic profiling, Ng (together with other synaptic injury–linked proteins such as SNAP-25) were elevated across the AD continuum and showed strong diagnostic discrimination, consistent with Ng aligning well with a damage-dominant dimension of synaptic pathology [17]. However, an injury-only dimension is unlikely to capture the key determinants of clinical progression, because patients with comparable synaptic damage may differ in residual synaptic and network-level adaptive capacity that helps stabilize circuit function. This is biologically plausible because neurons can engage local compensatory synaptic plasticity in response to synapse loss (e.g., enlargement of surviving spines and/or remodeling), even if the extent and clinical benefit of compensation vary across individuals [18].

Neuronal pentraxin-2 (NPTX2) is a compelling candidate to capture this complementary axis given its role in homeostatic regulation of cortical network dynamics and synaptic plasticity (Gómez de San José et al. 2021). CSF NPTX2 is reduced in AD dementia and correlated robustly with cognitive performance and hippocampal volume, and with a higher risk of progression from MCI in multiple cohorts [20–25]. Finally, among cognitively unimpaired individuals, lower CSF NPTX2 has been associated with a higher risk of incident MCI independent of core CSF AD biomarker levels [26].

This evidence makes CSF NPTX2 biologically plausible as a proxy of synaptic/network stability rather than merely of synapse loss/damage. In line with this, higher NPTX2 was observed in early-stage MCI-AD and was positively associated with bilateral precuneus glucose metabolism across MCI-AD subjects, supporting a link between CSF NPTX2 and preserved network-level functional integrity [27]. Complementarily, in preclinical AD, higher baseline NPTX2 was related to slower long-term cognitive decline after accounting for amyloid and tau [28]. Consistently, in a large at-risk cohort, higher CSF NPTX2 was associated with slower cognitive decline even after accounting for amyloid burden, while canonical synaptic injury markers showed the opposite pattern, further supporting the notion that NPTX2 captures a dimension distinct from synaptic damage and closer to cognitive resilience [29]. Recent large-scale proteomic studies further suggest that synaptic protein signatures, including NPTX2-related patterns, capture a biological dimension of cognitive resilience that is not fully explained by amyloid or tau burden [30]. Finally, in the BioFINDER-2 study, combining different dimensions with ratios of proteins increased in AD (e.g., SNAP-25 or 14-3-3ζ/δ, and neurogranin) over NPTX2 strengthened associations with cognitive decline and brain atrophy [17]. This view is consistent with the idea that the clinical trajectory may be better reflected jointly by synaptic injury markers and NPTX2-related network processes.

Together, these observations motivate a specific, testable hypothesis in biomarker-confirmed MCI-AD. Synaptic protein patterns may reveal clinically meaningful subgroups with distinct progression trajectories, reflecting inter-individual differences in downstream network vulnerability versus preserved capacity. Under this hypothesis, disproportionately low CSF NPTX2 in the context of synaptic injury and AD pathology may signal reduced ability to sustain activity-dependent network homeostasis, and thus a higher likelihood of short-to-midterm clinical progression. Conversely, relatively preserved or increased CSF NPTX2 could indicate greater synaptic/network adaptive capacity, potentially supporting prolonged cognitive stability within a compensatory framework.

A key challenge is to translate this idea into an interpretable metric while limiting redundancy across correlated synaptic markers. Because NPTX2 and Ng are both synaptic proteins, some covariation is expected, yet their relative weight may isolate prognostically relevant information. Ratio-based approaches provide a useful first step, but they inherently blend numerator and denominator effects, potentially limiting mechanistic interpretability [31]. An alternative, complementary strategy is to interpret CSF NPTX2 relative to its expected level given synaptic injury and core AD pathology, thereby focusing on whether NPTX2 is lower or higher than anticipated in this biological context.

In this study, we test this hypothesis in a cohort of typical MCI-AD patients defined by CSF biomarker-based criteria and followed longitudinally for clinical outcomes [1, 2]. We considered CSF Ng as our marker of synaptic injury and asked whether a specific CSF NPTX2 signal—interpreted in the context of synaptic injury and amyloid–tau burden—provides incremental prognostic information for cognitive decline. This signal may provide an operational proxy of synaptic resilience or compensatory network capacity, helping to clarify the biological and clinical meaning of variability in CSF synaptic protein levels across the MCI-AD spectrum.

## Methods

### Patients

We retrospectively selected 104 patients (42 females; age 72.6 ± 6.9 years, range 52.4–84.3 years; education 10.8 ± 4.4 years, 5–23 years; MMSE at baseline, MMSE BS, 26.1 ± 2.6, 19–30) with typical, high-likelihood MCI-AD [1] from a clinical database of 300 consecutive subjects diagnosed with MCI-AD at the Memory Clinic, University of Genoa (Italy), between May 2017 and January 2025. At the first assessment participants received neurological and general medical examinations as well as an extended neuropsychological battery, as previously described [32], detailing an amnestic profile (single- or multidomain). Activities of daily living and instrumental activities of daily living were preserved in all cases, as confirmed by clinical interview and standardized questionnaires. The Clinical Dementia Rating (CDR) was consistently 0.5, supporting a final diagnosis of amnestic MCI [33]. Routine blood tests and brain MRI excluded other causes of cognitive impairment. Patients with severe white matter hyperintensity burden on MRI (Fazekas grade 3) were excluded. All participants underwent lumbar puncture with assessment of CSF core AD biomarkers (amyloid-β42/amyloid-β40 ratio [Aβ42/40]; tau phosphorylated at threonine 181 [p-Tau181]; and total tau [t-Tau]), with an AD-compatible AT(N) profile in 98 patients and an A + T- profile for Alzheimer’s pathological changes in 6 patients [34]. To be included in the study, all individuals additionally had undergone an [^18^F]FDG PET scan within two months from baseline, showing a typical AD hypometabolic pattern [35]. The [^18^F]FDG PET positivity additionally allowed confirmation of AD in those 6 A + T− patients, as recommended within this specific diagnostic pathway [36]. A minimum clinical follow-up of one year was required to support diagnostic confirmation. No attrition occurred after cohort inclusion, as all patients had complete longitudinal clinical follow-up data, as per clinical routine.

Across the overall follow-up period (3.3 ± 2.1 years, 1–11 years), 55 patients progressed to dementia after 2.9 ± 1.9 years. Dementia progression was defined by a CDR score of 1 based on structured interviews with patients and informants. Using a 2-year threshold, we stratified MCI-AD into two stage subgroups. Differently from ADNI approaches that classified early/late MCI by severity of memory impairment [37, 38], we adopted a temporal criterion reflecting proximity to dementia, consistently with our previous works [27, 32]. Late MCI (LMCI, *n* = 30) was defined by progression to dementia within 2 years from baseline (1.5 ± 0.4 years), whereas early MCI (EMCI, *n* = 74) included individuals progressing after 2 years or remaining stable at the MCI stage throughout observation.

### CSF biomarker measures

CSF sampling and pre-analytical handling followed standard operating procedures as described in Supplementary Methods. We measured core AD CSF biomarkers (Aβ42, Aβ40, p-Tau181, and t-Tau) using the Lumipulse G600 II^®^ fully automated chemiluminescent enzyme immunoassay platform (Fujirebio Europe, Ghent, Belgium). Manufacturer cartridge cut-offs were locally verified in our laboratory: Aβ42/40 ratio 0.069, pTau181 56.5 pg/mL, and t-Tau 404 pg/mL [27, 32]. On the same CSF aliquots, CSF NPTX2 and neurogranin (trunc P75, Ng) were quantified with commercially available ELISA kits (INNOTEST, Fujirebio Europe, Ghent, Belgium; and Euroimmun, Lübeck, Germany, respectively). Concentrations are reported in pg/mL. Intra-assay coefficients of variation were < 10% on average, and inter-assay coefficients of variation were < 15% for all assays.

For CSF biomarker comparisons, the same proteins were measured in a control group of 43 age- and sex-matched subjects with other non-dementing (OND) conditions who underwent CSF testing for diagnostic and research purposes [27, 32]. This group comprised 18 patients with normal pressure hydrocephalus, 9 with cerebrovascular disease, 7 with amyotrophic lateral sclerosis without cognitive impairment, 4 with inflammatory disorders of the peripheral or central nervous system, 3 worried-well individuals with no cognitive disorder after extensive work-up, and 2 healthy subjects undergoing lumbar puncture for orthopedic surgery. Cognitive status was verified by MMSE > 27 and CDR = 0, and AD pathology was excluded by normal CSF Aβ42/40 ratio and pTau181 values [34].

### Statistical analysis 

Demographic and clinical variables were compared between EMCI and LMCI using two-tailed t-tests or Wilcoxon rank-sum tests, as appropriate, with correction for multiple comparisons. Categorical variables were assessed with Fisher’s exact test. CSF biomarker levels across EMCI, LMCI, and OND were evaluated with Kruskal–Wallis test and Benjamini–Hochberg FDR-corrected post-hoc comparisons. Associations among CSF biomarkers and with demographic/clinical variables were assessed by age-adjusted partial Spearman correlations.

To explore whether the distinct synaptic information provided by Ng and NPTX2 identifies subgroups with different likelihood of short-term clinical progression—i.e., LMCI progressing to dementia within 2 years vs. later progressors (EMCI)—CSF neurogranin and NPTX2 were dichotomized using sample medians to define four Ng–NPTX2 quadrants (low < median; high ≥ median). The distribution of progression status (EMCI vs. LMCI) across quadrants was tested using a 2 × 4 chi-square test of independence. Participants were additionally collapsed by CSF NPTX2 low vs. high and by CSF Ng low vs. high, and LMCI frequencies were compared using two-sided Fisher’s exact test, reporting odds ratios (ORs).

CSF biomarkers were z-standardized within the MCI cohort. AD pathological burden was summarized as a single index capturing the amyloid–tau load in one metric, defined as ‘AD burden’ and computed as z(pTau) divided by z(Aβ42/40). Aβ42/40 ratio was preferred to Aβ42 as a metric of amyloidosis considering the higher accuracy this ratio provides in the classification of AD [39]. We derived an age-adjusted residual of CSF NPTX2 (hereafter, Res_NPTX2) as the residual from an ordinary least squares (OLS) model regressing z(NPTX2) on z(Ng), AD burden, and age. Therefore, Res_NPTX2 was defined as the age-adjusted residual of CSF NPTX2 conditional on standardized Ng and AD burden and was used as the operational readout within the proposed compensatory framework. Variance inflation factors (VIFs) were calculated to assess multicollinearity among.

predictors included in the residualization model. Res_NPTX2 was subsequently compared in EMCI vs. LMCI, then tested in OLS regression models against clinical outcomes, i.e., MMSE BS, MMSE at the last available follow-up visit, MMSE FU, and their difference (ΔMMSE, i.e., [MMSE BS-MMSE FU] where positive values indicate higher cognitive decline). To account for variable cognitive status at diagnosis and observation windows, OLS regression was re-tested with ΔMMSE as the dependent variable and Res_NPTX2 as the predictor, adjusting for MMSE BS and FU duration in years. As a sensitivity analysis, we tested whether baseline z(NPTX2) or z(Ng) predicted MMSE decline over time by fitting OLS models with ΔMMSE as outcome, adjusting for MMSE BS and FU duration, entering z(NPTX2) and z(Ng) either separately or jointly. As an additional analysis, receiver operating characteristic (ROC) curves were used to compare the ability of CSF Ng, CSF NPTX2, and Res_NPTX2 to discriminate short-term progression status, defined as LMCI versus EMCI.

Time-to-progression was analyzed using Cox proportional-hazards regression with Res_NPTX2 as predictor. Time was defined from baseline assessment to clinical progression to AD dementia. Participants who did not progress were censored at their last available FU visit. Effect sizes are reported as hazard ratios (HRs) with 95% confidence intervals (CIs). The primary model tested Res_NPTX2 as predictor. Sensitivity analyses evaluated (i) MMSE BS alone or (ii) MMSE BS plus Res_NPTX2 to test whether the association between Res_NPTX2 and time-to-progression was robust to adjustment for baseline cognitive status. Nested models were compared using partial likelihood ratio (LR) tests (difference in − 2 log partial likelihood), reporting LR χ² and p-values.

Participants were categorized into tertiles of Res_NPTX2 (Low ≤ − 0.38; Mid − 0.38 to 0.09; High > 0.09). Time-to-conversion to AD dementia was analyzed using Kaplan–Meier curves and compared with the log-rank test; an ordered log-rank test for trend was used to assess monotonic patterns across tertiles. The Gehan–Breslow–Wilcoxon test was additionally reported as an early-event–weighted sensitivity check. Non-progressors were censored at their last available follow-up visit. Hazard ratios were estimated using Cox proportional-hazards regression with tertiles modeled categorically using dummy coding (Low as reference) to obtain Mid vs. Low and High vs. Low without assuming linearity. Post-hoc pairwise log-rank comparisons (Low–Mid, Low–High, Mid–High) were performed with Holm–Šidák correction.

As a sensitivity analysis focused on early progression, we applied 2-year administrative censoring by truncating follow-up at 2 years and coding as events only progression to dementia occurring within 2 years with all other observations censored. We additionally performed a 2-year landmark analysis restricted to participants event-free at 2 years, re-indexing time from the 2-year landmark and modeling progression risk beyond year 2.

### Exploratory two-layer compensation framework

We defined OND-referenced NPTX2 bands using the distribution of CSF NPTX2 measured in the OND group (mean ± SD 832.9 ± 526.8 pg/mL). Based on fixed thresholds at ± 0.5 SD around the OND mean we classified MCI-AD patients as: Low if NPTX2 ≤ 569.5 pg/ml, High if NPTX2 ≥ 1096.3 pg/mL, and Intermediate otherwise. These OND-referenced bands (hereafter, NPTX2_OND_) were used as the first layer to be combined with the statistically isolated NPTX2-related residual component (Res_NPTX2). OND anchoring was applied only to CSF NPTX2 to provide an external, clinically interpretable reference, whereas Res_NPTX2 was derived within the MCI-AD cohort to quantify the NPTX2-specific component conditional on synaptic damage (Ng), AD burden, and age, as previously described. We then defined compensation phenotypes as (i) compensation-preserved with High NPTX2_OND_ & High Res_NPTX2 > 0.09, (ii) compensation-failure (Low NPTX2_OND_ & Low Res_NPTX2 ≤ − 0.38), and (iii) intermediate (all others). These labels were used to operationalize the compensatory framework and should be interpreted as prognostic biomarker profiles rather than direct evidence of active compensatory mechanisms. Group differences in the frequency of short-term conversion (LMCI ≤ 2 years) were tested by Chi-squared test.

Statistical analysis utilized Jamovi (Version 2.6) and Prism (version 10.2.1, GraphPad Software, San Diego, California, USA), with *p* < 0.05 significance threshold.

### [^18^F]FDG PET acquisition and statistical analysis

We obtained [^18^F]FDG PET scans at baseline following the current European Association of Nuclear Medicine (EANM) guidelines [40, 41]. The methodology for acquisition and image pre-processing is consistent with prior research [27, 32, 42] and detailed in Supplementary materials.

Following preprocessing, the smoothed images of the MCI-AD patients underwent a voxel-based group analysis (VBA) using the Statistical Parametric Mapping (SPM12) software, exploring the whole brain without any a priori hypothesis of involved brain regions, including:

1. The comparison between the scans of the patients and a healthy control (HC) group of 40 age-, sex- and education-matched volunteers [27, 32, 42] (two-sample t-test) with age as nuisance. Relatively hypometabolic regions in patients were labelled as AD-related volumes of interest (MCI-AD VOIs).
2. The comparison between EMCI and LMCI patients (two-sample t-test; nuisance: age) to obtain those regions with different brain metabolism in later MCI stage approaching dementia than in the early MCI sub-stage.
3. The multiple linear regression analysis to identify a CSF NPTX2-related VOI using [^18^F]FDG regional uptake as dependent variable, CSF NPTX2 values as covariate, and age as a nuisance. CSF Ng as a confounder was subsequently incorporated for exploratory purposes. Similarly, a multiple linear regression analysis was performed to identify Res_NPTX2-related VOIs with age as a nuisance.

Voxel values were normalized according to cerebellar cortex activity following.

the approach described in previous work [27, 42]. This normalization enhances the detection of cortical abnormalities by leveraging the supposedly unaffected cerebellum in AD. For the VBA, CSF NPTX2 values were log-transformed due to right-skewed distribution (*p* < 0.001 at Lilliefors test), enabling linear models. The analysis was conducted exclusively on the MCI-AD group.

A 0.8 grey matter threshold mask was applied and statistical maps were thresholded at voxel-level *p* < 0.002 uncorrected, with cluster-level family-wise error correction at *p* < 0.05 and a minimum cluster extent of 100 voxels.

Cluster coordinates were processed using GingerALE and Talairach Client software packages to align them with the Brodmann-classified atlas.

## Results

### Clinical and CSF biomarker data

Table 1 summarizes the clinical-demographic features and CSF biomarker values for MCI-AD patients. Participants were stratified into EMCI (*n* = 74) and LMCI (*n* = 30) based on progression within 2 years. Compared to EMCI, the LMCI subgroup showed lower MMSE BS (24.6 ± 2.7 vs. 26.7 ± 2.3; *p* < 0.001) and MMSE FU (16.0 ± 4.6 vs. 21.8 ± 5.5; *p* < 0.001), and higher ∆MMSE (8.8 ± 4.5 vs. 4.9 ± 5.2; *p* < 0.001).

**Table 1:** Clinical characteristics and CSF biomarkers levels of MCI-AD patients

Demographical / clinical variables	MCI-AD (*n* = 104)	EMCI (*n* = 74)	LMCI (*n* = 30)		*p* value^#^	*p*_ADJ_ value*
Age (years)	72.6 ± 6.9	72.9 ± 6.4	71.7 ± 8.2		0.669	> 0.99
Sex (M: F)	42:62	33:41	9:21		0.192^§^	
Education	10.8 ± 4.4	11.0 ± 4.2	10.5 ± 4.8		0.548	> 0.99
MMSE BS	26.1 ± 2.6	26.7 ± 2.3	24.6 ± 2.7		**< 0.001**	**< 0.001**
MMSE FU	20.1 ± 5.8	21.8 ± 5.5	16.0 ± 4.6		**< 0.001**	**< 0.001**
∆ MMSE	6.0 ± 5.3	4.9 ± 5.2	8.8 ± 4.5		**< 0.001**	**< 0.001**

**CSF biomarkers**	**MCI-AD (*n*** **= 104)**	**EMCI (*n*** **= 74)**	**LMCI (*n*** **= 30)**	**OND (*n*** **= 43)**	**p-value** ^**¶**^	**Post-hoc comparisons** ^**‡**^
Aβ42/40 ratio	0.046 ± 0.010	0.046 ± 0.010	0.048 ± 0.010	0.098 ± 0.015	**< 0.0001**	EMCI < OND *p* < 0.001 LMCI < OND *p* < 0.001
pTau181 (pg/ml)	108.7 ± 58.3	110.0 ± 62.11	105.4 ± 48.6	26.2 ± 9.1	**< 0.0001**	EMCI > OND *p* < 0.001 LMCI > OND *p* < 0.001
t-Tau (pg/ml)	723.8 ± 367.8	728.2 ± 377.5	712.8 ± 348.3	233.3 ± 106.4	**< 0.0001**	EMCI > OND *p* < 0.001 LMCI > OND *p* < 0.001
NPTX2 (pg/ml)	1022.3 ± 527.1	1141.2 ± 559.0	729.0 ± 274.2	832.9 ± 526.8	**0.001**	EMCI > OND *p* < 0.001 EMCI > LMCI *p* < 0.001
Ng (pg/ml)	636.6 ± 328.8	676.9 ± 337.1	537.3 ± 288.9	248.4 ± 156.4	**< 0.0001**	EMCI > OND *p* < 0.001 LMCI > OND *p* < 0.001

*Abbreviations*: *MCI-AD* Mild cognitive impairment due to Alzheimer’s disease, *EMCI* Early mild cognitive impairment, *LMCI* Late mild cognitive impairment, *F* Female, *M* Male, *MMSE* Mini Mental State Examination, *BS* Baseline, *FU* Follow-up, *CSF* Cerebrospinal fluid, *Aβ42/40* Ratio amyloid *Aβ1-42* Normalized for amyloid Aβ1-40, *pTau181* Tau phosphorylated at threonine 181, *t-Tau *Total Tau, *NPTX2* Neuronal Pentraxin 2, *Ng* Neurogranin

^§^p-values for Fisher’s exact test

^#^p-values for Wilcoxon rank-sum test between EMCI and LMCI subgroups

^*^.Bonferroni-Dunn adjusted p-value

^**¶**^p-values for Kruskal–Wallis comparing EMCI, LMCI, and OND subgroups

‡p-values for Benjamini–Hochberg FDR-corrected post-hoc comparisons

Mean ± SD are shown. Significant values are in bold

**Fig. 1:**
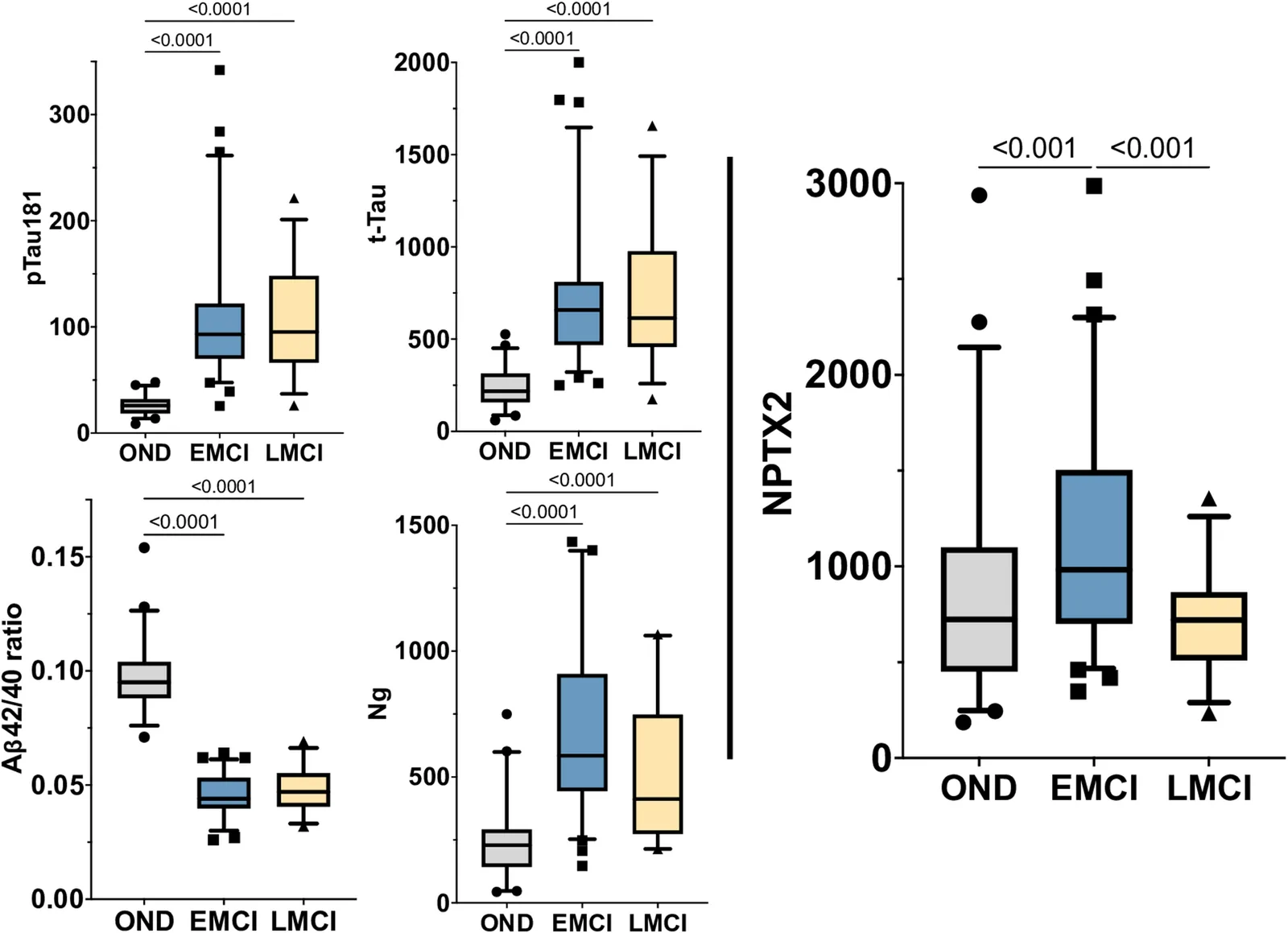
CSF AD core biomarkers and synaptic proteins across OND, EMCI and LMCI. Box-and-whisker plots show baseline CSF concentrations of Aβ42/40 ratio, p-Tau181, t-Tau, neurogranin (Ng), and neuronal pentraxin-2 (NPTX2) in non-dementing controls (OND) and patients with mild cognitive impairment due to Alzheimer’s disease (MCI-AD) stratified into early (EMCI) and late (LMCI) stage according to a 2-year progression status. Boxes indicate the median and interquartile range (IQR), and the whiskers indicate the 5% to 95% percentiles. Any statistically significant FDR-corrected p-values are displayed as numbers

In group analyses, CSF AD core biomarkers and Ng differed in EMCI and LMCI compared with OND (*p* < 0.001), but not one with respect to another. Conversely, CSF NPTX2 differed among groups (*p* = 0.001) and in post-hoc analyses higher NPTX2 was observed in EMCI compared to LMCI and OND (*p* = 0.001 for both) (Table 1; Fig. 1).

In age-adjusted Spearman analyses of CSF biomarkers in the MCI-AD cohort, the Aβ42/40 ratio exhibited a negative correlation with both pTau181 (*p* = 0.003, *r*=-0.29) and t-Tau (*p* = 0.024, *r*=-0.22), as expected. CSF NPTX2 and Ng were strongly positively correlated (*p* < 0.001, *r* = 0.67) with each other and both exhibited significant associations with core AD biomarkers (all *p* ≤ 0.002). With respect to cognition, CSF NPTX2 correlated positively with MMSE BS (*p* = 0.050, *r* = 0.19) and more strongly with MMSE FU (*p* < 0.001, *r* = 0.38). MMSE BS was associated with years of education (*p* = 0.006) and—expectedly—with MMSE FU (*p* < 0.001), which in turn significantly correlated with pTau181 (*p* = 0.003) and t-tau (*p* = 0.018) (Table S1).

**Fig. 2:**
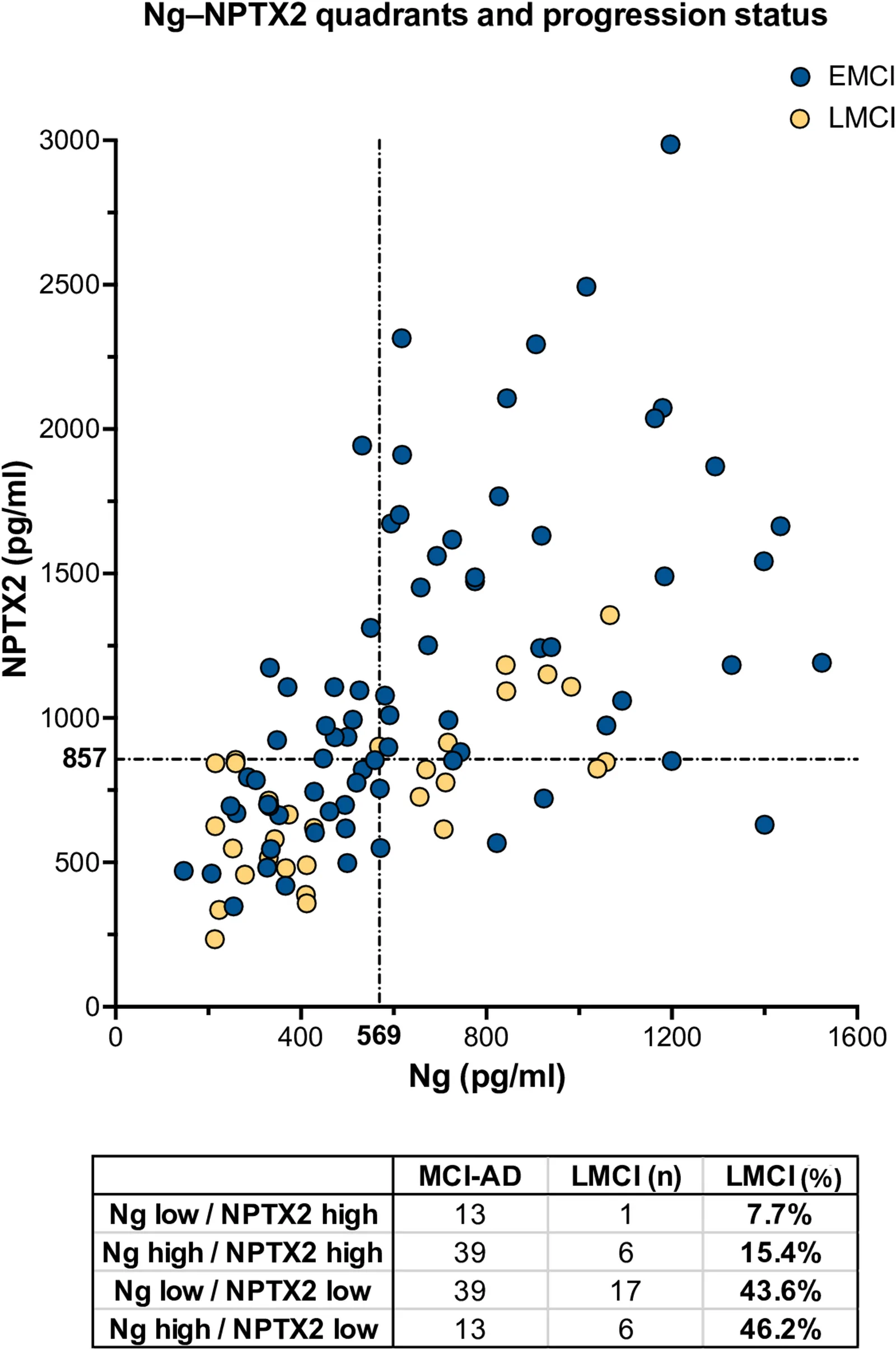
Ng–NPTX2 quadrants by progression group (EMCI vs. LMCI). Scatterplot of baseline CSF NPTX2 versus CSF Ng (pg/mL), with points colored by progression group (EMCI vs. LMCI). Dashed vertical and horizontal lines indicate the cohort medians (Ng = 569 pg/mL; NPTX2 = 857 pg/mL), defining four median-split quadrants (Ng low/NPTX2 low, Ng low/NPTX2 high, Ng high/NPTX2 low, Ng high/NPTX2 high). The table reports the number of participants in each quadrant and the corresponding proportion classified as LMCI

After dichotomizing CSF Ng and NPTX2 values at the sample medians (569.1 pg/ml and 857.1 pg/ml, respectively) and assigning each participant to one of four Ng–NPTX2 quadrants (low < median; high ≥ median), the proportion of LMCI differed markedly across quadrants: 7.7% in Ng low/NPTX2 high (1/13), 15.4% in Ng high/NPTX2 high (6/39), 43.6% in Ng low/NPTX2 low (17/39), and 46.2% in Ng high/NPTX2 low (6/13) (Fig. 2), yielding a significant association between quadrants and progression status (χ²(3) = 12.31, *p* = 0.006) (Supplementary Fig. 1s). Together, this median-split framework indicated that proximity to progression was better captured by CSF NPTX2. Collapsing participants by CSF NPTX2 showed substantially higher LMCI frequency in the low-NPTX2 group (23/52, 44.2%) compared with the high-NPTX2 group (7/52, 13.5%; *p* = 0.001, 95% CI 1.94–13.40, OR = 5.10). By contrast, collapsing by CSF Ng did not reveal a significant difference (low Ng: 18/52, 34.6% vs. high Ng: 12/52, 23.1%; *p* = 0.279).

The linear model of baseline z(NPTX2) on z(Ng), AD burden, and age was significant (F(3, 100) = 16.30, *p* < 0.0001) and explained ~ 33% of the variance in z(NPTX2) (R²=0.33; adjusted R²=0.31). z(Ng) was the only significant predictor (β = 0.62, 95% CI 0.40–0.83; *p* < 0.0001), whereas AD burden (β=−0.07; *p* = 0.507) and age (β=−0.002; *p* = 0.828) were not associated with z(NPTX2). Variance inflation factors were low for all predictors (z(Ng) = 1.76, AD burden = 1.74, age = 1.02), indicating negligible multicollinearity in the residualization model (Table 2A).

**Table 2:** Multivariable linear regression models

Panel A. Predicting z(NPTX2) (model used to derive Res_NPTX2)							
Model fit	*N*	DF (model, residual)	F		*p*-value	*R*²	Adj *R*²
	104	3, 100	16.30		**< 0.0001**	0.33	0.31
**Predictor**	**β**	**SE**	**95% CI**	**t (df = 100)**	**p-value**	**Partial R²**	**VIF**
Intercept	0.189	0.872	-1.54 to 1.92	0.22	0.829	—	—
z(Ng)	0.619	0.109	0.40 to 0.83	5.69	**< 0.0001**	0.24	1.76
AD burden	-0.072	0.108	-0.29 to 0.14	0.66	0.508	0.004	1.74
Age	-0.0026	0.0120	-0.03 to 0.02	0.22	0.828	0.0005	1.02

**Panel B. Simple linear regression models between Res_NPTX2 and cognitive outcomes**							
**Predictor**	**Outcome**	**β (slope)**	**95% CI**		**p-value**	**R²**	
Res_NPTX2	MMSE BS	+ 0.82	0.22 to 1.42		**0.008**	0.07	
Res_NPTX2	MMSE FU	+ 2.54	1.24 to 3.85		**0.0002**	0.13	
Res_NPTX2 (BS − FU)	ΔMMSE (BS − FU)	−1.76	−2.98 to − 0.54		**0.005**	0.07	

**Panel C. Predicting ΔMMSE (BS-FU)**							
**Model fit**	**N**	**DF (model**,** residual)**	**F**		**p-value**	**R²**	**Adj R²**
	104	3, 100	10.59		**< 0.0001**	0.24	0.22
**Predictor**	**β**	**SE**	**95% CI**	**t (df = 100)**	**p-value**	**Partial R²**	**VIF**
Intercept	0.88	4.83	-8.69 to 10.46	0.18	0.856	—	—
Res_NPTX2	-1.88	0.58	-3.04 to -0.74	-3.23	**0.002**	0.09	1.07
MMSE_BS	0.068	0.188	-0.305 to 0.440	0.36	0.720	0.001	1.11
FU (years)	1.03	0.23	0.58 to 1.48	4.52	**< 0.0001**	0.17	1.04

Two-sided p-values are reported; 95% confidence intervals are shown

All models were fit by ordinary least squares (OLS). β values are unstandardized regression coefficients (slopes). z() indicates within-cohort z-transformation. In Panel A, the model predicts z(NPTX2) from z(Ng), AD burden, and age; Res_NPTX2 is defined as the residual of this model (observed minus model-predicted z(NPTX2)). In Panels B and C, ΔMMSE is defined as MMSE baseline minus MMSE follow-up; therefore, higher ΔMMSE indicates greater cognitive decline. FU (years) is the follow-up duration in years. Partial R² reflects the unique variance explained by each predictor (e.g., SS_effect/(SS_effect+SS_error)). VIF values quantify multicollinearity (lower values indicate negligible collinearity)

*Abbreviations:* *AD* Alzheimer’s disease, *BS* Baseline, *CI* Confidence interval, *df* Degrees of freedom, *FU* Follow-up, *MMSE* Mini-Mental State Examination, *Ng* Neurogranin, *NPTX2* neuronal pentraxin-2, *Res_NPTX2* Residual NPTX2 component (as defined above), *SE* Standard error, *VIF* Variance inflation factor

**Fig. 3:**
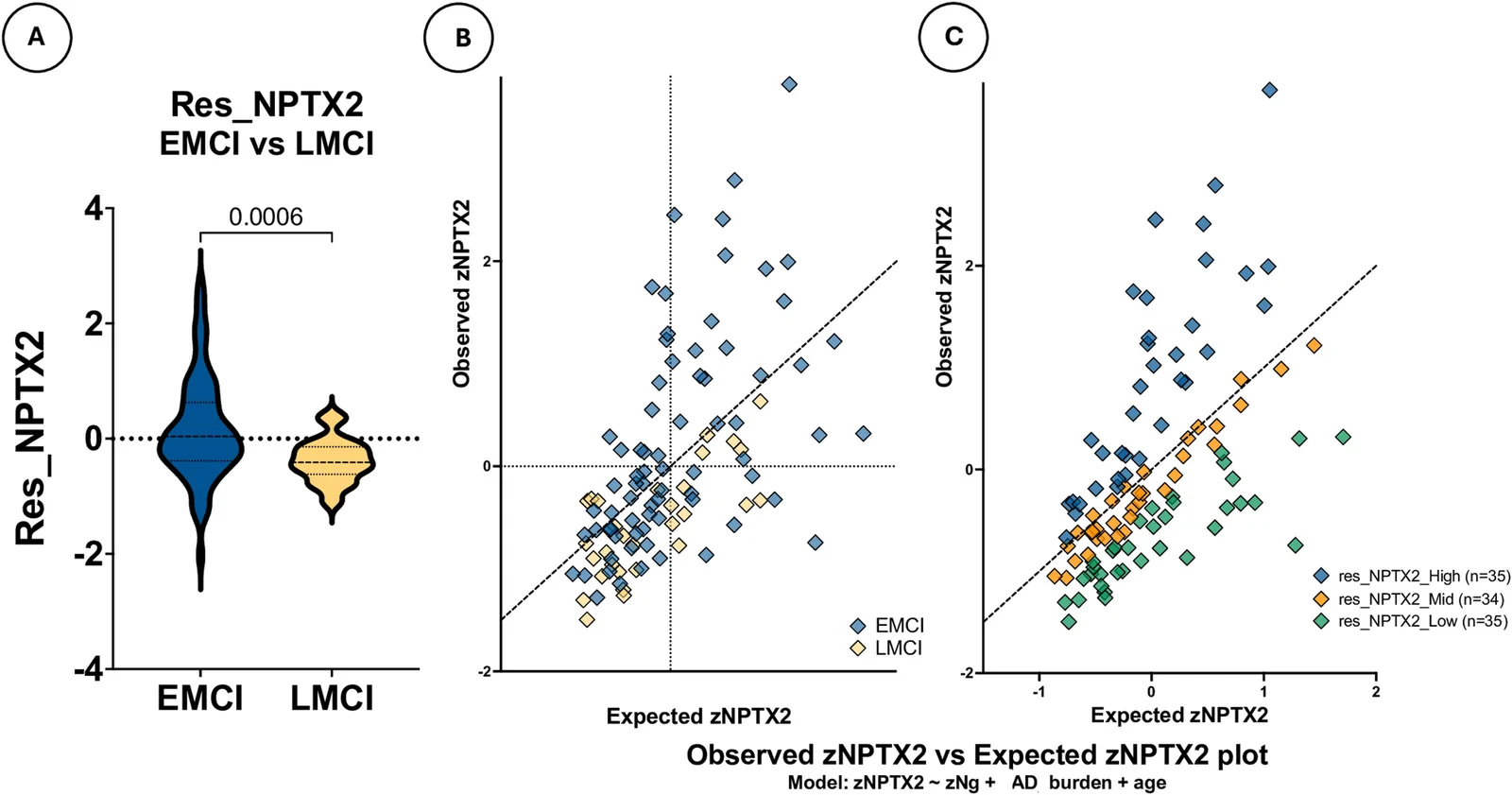
**A** Res_NPTX2 in EMCI vs. LMCI. Violin plots show the distribution of Res_NPTX2 in EMCI and LMCI. The horizontal reference line at 0 indicates the expected value (no deviation from model prediction). Central dashed line indicates the median and dotted lines indicate the interquartile range. Group difference was tested with a two-sided Wilcoxon rank-sum test (p-value shown as number). **B** Observed vs expected z(NPTX2) by progression group. Scatterplot of observed baseline z(NPTX2) (y-axis) versus expected z(NPTX2) values (x-axis), which are model-predicted from z(NPTX2) ~ z(Ng) + AD burden + age. The dashed identity line (y = x) denotes perfect agreement between observed and expected values; vertical deviations from this line correspond to the residual (Res_NPTX2). Diamonds are color-coded by progression status (EMCI vs LMCI). **C** Observed vs expected z(NPTX2) stratified by Res_NPTX2 tertiles. Same plot as in panel B, with diamonds color-coded by tertiles of Res_NPTX2 (High, Mid, Low). Participants in the highest Res_NPTX2 tertile lie predominantly above the identity line (positive residual: higher-than-expected NPTX2 given Ng, AD burden and age), whereas those in the lowest tertile lie predominantly below the identity line

Res_NPTX2 values were significantly higher in EMCI compared with LMCI (*p* = 0.0006) (Fig. 3A). At the individual level, EMCI showed more frequently positive deviations of observed from expected zNPTX2, whereas LMCI negative deviations (Fig. 3B). Together, these panels support Res_NPTX2 as an interpretable residual-based metric capturing a putative, statistically isolated NPTX2-related adaptive-capacity component, subsequently tested against clinical outcomes within the proposed compensatory framework.

**Fig. 4:**
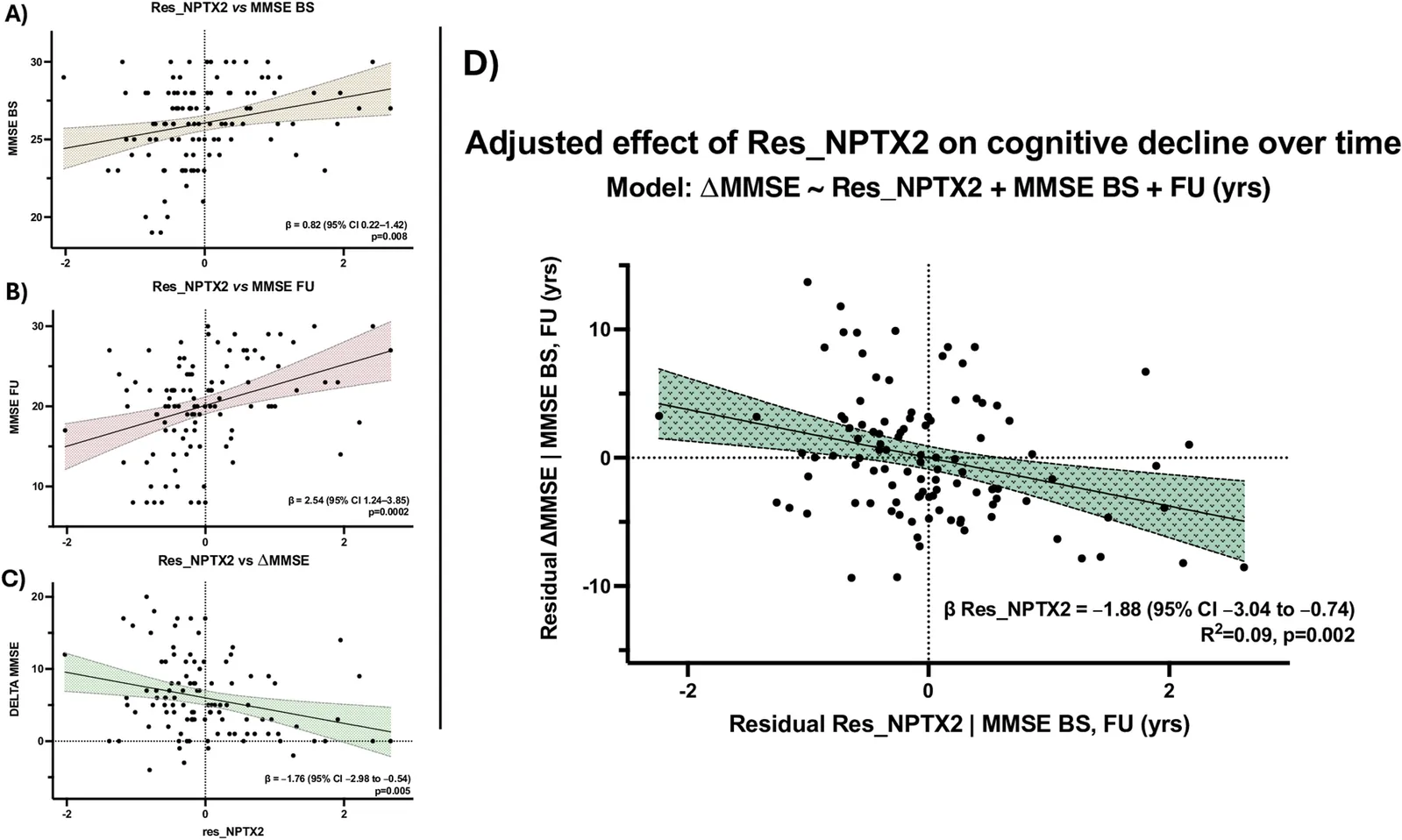
Res_NPTX2 and cognitive decline. **A** Scatterplot of baseline MMSE (MMSE_BS) versus baseline Res_NPTX2, with least-squares regression line and 95% confidence band (β=0.82; 95% CI 0.22–1.42; p=0.008). **B** Scatterplot of follow-up MMSE (MMSE FU) versus baseline Res_NPTX2 (β=2.54; 95% CI 1.24–3.85; *p*=0.0002). **C** Scatterplot of cognitive decline (ΔMMSE = MMSE BS – MMSE FU; higher values indicate greater decline) versus baseline Res_NPTX2 (β=−1.76; 95% CI −2.98 to −0.54; p=0.005). **D** Added-variable (partial regression) plot illustrating the adjusted association between Res_NPTX2 and cognitive decline in the multivariable model ΔMMSE ~ Res_NPTX2 + MMSE BS + FU duration (years). The y-axis shows residualized ΔMMSE after removing effects of MMSE BS and FU duration; the x-axis shows residualized Res_NPTX2 after removing the same covariates. The fitted line and 95% confidence band correspond to the adjusted Res_NPTX2 coefficient (β=−1.88; 95% CI −3.04 to −0.74; *p*=0.002; partial R²≈0.09). Dotted reference lines indicate zero residuals

Higher Res_NPTX2 was associated with higher MMSE BS (β = 0.82; 95% CI 0.22–1.42; R²=0.07; *p* = 0.008) and MMSE FU (β = 2.54; 95% CI 1.24–3.85; R²=0.13; *p* = 0.0002) (Fig. 4A-B). Moreover, higher Res_NPTX2 predicted less ΔMMSE (β=−1.76; 95% CI − 2.98 to − 0.54; R²=0.07; *p* = 0.005), even after adjusting for MMSE BS and FU duration (β = −1.88; 95% CI − 3.04 to − 0.73; *p* = 0.002; model R² = 0.24) (Fig. 4C-D and Table 2B-2C).

In the sensitivity analysis testing whether baseline z(NPTX2) and z(Ng) were associated with ΔMMSE when modelled directly (adjusting for baseline MMSE and follow-up duration), z(NPTX2) but not z(Ng) predicted less MMSE decline (z(NPTX2): β= −1.55 points per SD; 95% CI − 2.50 to − 0.61; *p* = 0.0015; z(Ng): β= −0.43; 95% CI − 1.39 to 0.53; *p* = 0.38). When z(NPTX2) and z(Ng) were entered simultaneously in the model, z(NPTX2) remained independently associated with less decline (β= −1.95; 95% CI − 3.10 to − 0.80; *p* = 0.001), whereas z(Ng) did not show an independent effect (*p* = 0.24) (Supplementary Table S2). In additional ROC sensitivity analyses for discriminating LMCI from EMCI, CSF Ng showed only modest discrimination (AUC = 0.63), whereas CSF NPTX2 and Res_NPTX2 showed higher and closely comparable performance (AUC = 0.73 and AUC = 0.71, respectively) (Supplementary Figure S3).

In a univariable Cox proportional-hazards model, higher Res_NPTX2 was associated with a significantly lower hazard of conversion to AD dementia (β(SE) − 0.46(0.17), HR = 0.63 per 1-point increase, 95% CI 0.45–0.88; *p* = 0.007). In sensitivity analyses, MMSE BS was strongly protective (HR = 0.78, 95% CI 0.71–0.87; *p* < 0.0001). Importantly, adding Res_NPTX2 to the MMSE BS model improved model fit (ΔAIC = − 3.7, LR χ²(1) = 5.7; *p* = 0.017), and Res_NPTX2 remained independently associated with lower hazard of conversion (HR = 0.64, 95% CI 0.43–0.93; *p* = 0.025) (Supplementary Table S3). Visual inspection of scaled Schoenfeld residuals did not indicate an appreciable time-dependent trend, supporting the proportional hazards assumption.

**Fig. 5:**
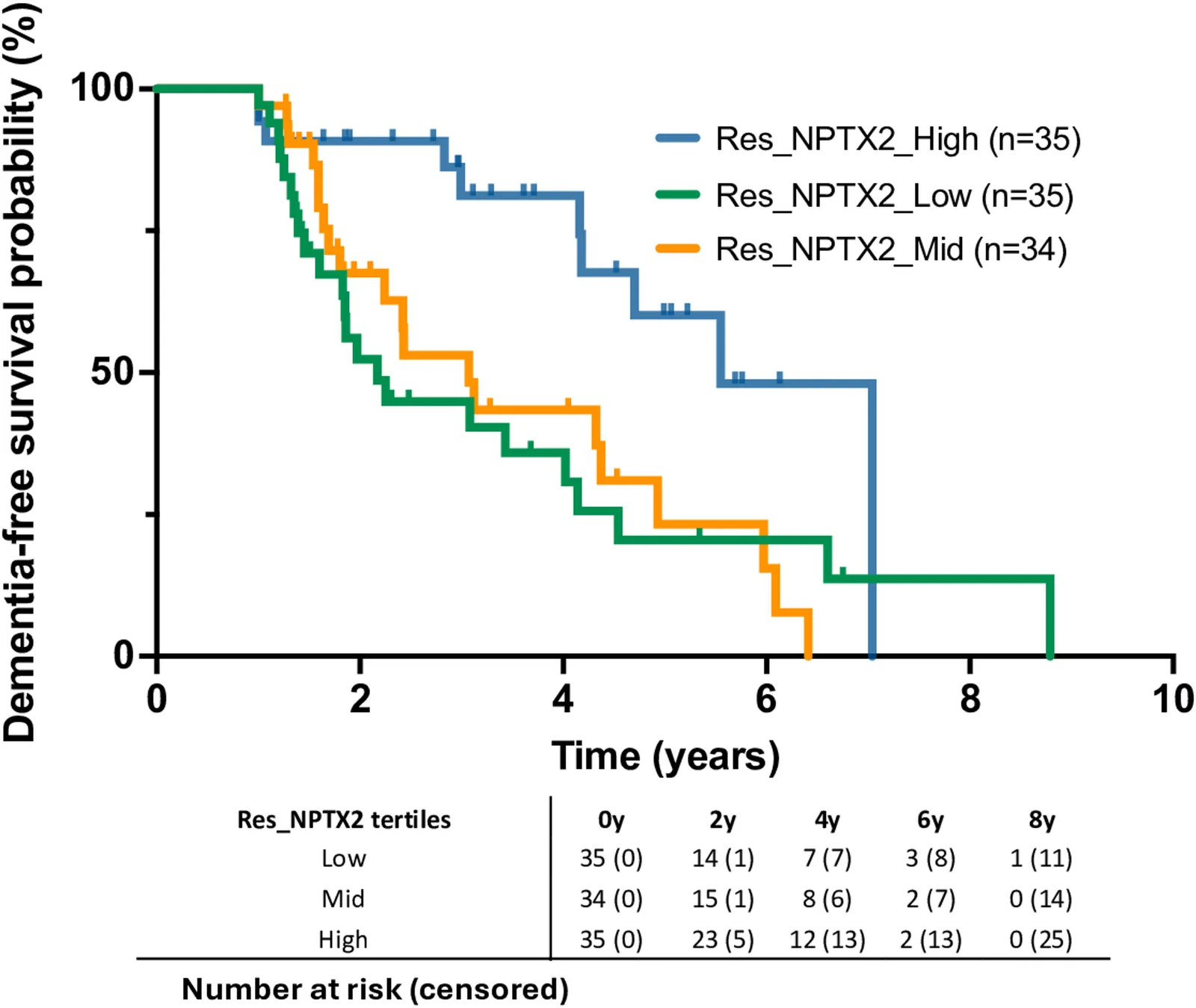
Kaplan–Meier curves for time-to-conversion to AD dementia across tertiles of Res_NPTX2. Kaplan–Meier curves show the probability of remaining free from AD dementia progression across tertiles of Res_NPTX2 defined within the MCI cohort: Low (Res_NPTX2 ≤ − 0.38; *n* = 35), Mid (− 0.38 < Res_NPTX2 ≤ 0.09; *n* = 34), and High (Res_NPTX2 > 0.09; *n* = 35). Tick marks indicate censoring at the last available follow-up visit. The table below the plot reports the number at risk (number censored) at 0, 2, 4, 6 and 8 years. Survival curves differed significantly across tertiles (log-rank Mantel–Cox χ²=9.426, df = 2, *p* = 0.009), with evidence of an ordered trend (logrank test for trend χ²=7.858, df = 1, *p* = 0.005). An early-event–weighted Gehan–Breslow–Wilcoxon test was also significant (χ²=9.023, df = 2, *p* = 0.011)

In survival analysis, Res_NPTX2 tertiles were associated with time-to-progression to AD dementia (Fig. 5, details in Supplementary Table S4). Kaplan–Meier curves differed significantly across tertiles (log-rank Mantel–Cox χ²=9.43, *p* = 0.009), with evidence of a monotonic trend from Low to High Res_NPTX2 tertile (log-rank test for trend χ²=7.86, *p* = 0.005). In Cox proportional-hazards models treating tertiles categorically (Low as reference), the High tertile showed a markedly reduced hazard of progression compared with Low (HR = 0.35, 95% CI 0.16–0.74; *p* = 0.006), whereas Mid did not differ from Low (HR = 1.04 95% CI 0.57–1.90; *p* = 0.897). Post-hoc pairwise log-rank comparisons supported this pattern: High differed from both Low and Mid, remaining significant after Holm–Šidák correction (*p* = 0.006 for both), while Mid vs. Low was not significant (*p* = 0.965). Overall, these results suggest that the survival difference across tertiles is driven primarily by the High Res_NPTX2 group rather than a uniform linear shift across all three groups. The Gehan–Breslow–Wilcoxon test of Kaplan–Meier curves - which places greater weight on early events - was also significant (χ²=9.02, *p* = 0.011), supporting concentrating secondary analyses on early disease course, i.e., within 2 years.

Under the 2-year administrative censoring, the tertile separation persisted and was concentrated early, i.e., within the first 2 years (Cox trend HR = 0.50 per tertile, *p* = 0.006; High vs. Low HR = 0.18, *p* = 0.008), whereas after a 2-year landmark among participants event-free at 2 years (*n* = 50), the association attenuated and was no longer significant (trend HR = 0.89, *p* = 0.624) (Supplementary Table S4). Landmark sample sizes/subsequent events were 12/7, 15/11, and 23/7 in the Low, Mid, and High tertiles, respectively. A formal assessment based on the association between scaled Schoenfeld residuals and log-transformed time was not significant (*p* = 0.077), providing no formal evidence of violation of the proportional-hazards assumption.

### Exploratory two-layer compensation framework

Based on the OND-anchored clinical translation level, 18 out of 104 (17.3%) MCI-AD patients were classified as Low NPTX2_OND_, 51/104 (49.0%) as Mid NPTX2_OND_, and 35/104 (33.7%) as High NPTX2_OND_. The proportion of short-term progressors (LMCI) increased across bands (High NPTX2_OND_: 4/35, 11.4%; Mid NPTX2_OND_: 17/51, 33.3%; Low NPTX2_OND_: 9/18, 50.0%), yielding a significant association between NPTX2_OND_ band and short-term progression status (χ²(2) = 9.60, *p* = 0.008). The two-layer (NPTX2_OND_ + Res_NPTX2) compensation-related phenotypes sharpened clinical separation and revealed a structured pattern. Collapsing cells into three compensation phenotypes produced clear risk gradients: High NPTX2_OND_ + High Res_NPTX2 (compensation-preserved) contained 24 patients and no short-term progressors 0/24 LMCI (0.0%); Low NPTX2_OND_ + Low Res_NPTX2 (compensation-failure) contained 15 patients with the highest progression frequency (8/15 LMCI, 53.3%), and the intermediate phenotype with the other combinations (*n* = 65) showed 22/65 LMCI ≤2y (33.8%) (χ²(2) = 14.90, *p* = 0.0006) (Supplementary Figure S2). Over the full follow-up window, the same phenotypes remained clinically distinct: progression to dementia occurred in 2/24 (8.3%) of compensation-preserved, 42/65 (64.6%) of Intermediate, and 11/15 (73.3%) of compensation-failure (χ²(2) = 25.22, *p* < 0.001) (Supplementary Table S5).

### [^18^F]FDG PET Voxel-Based Analysis (VBA)

Four relatively hypometabolic MCI-AD VOIs encompassed bilaterally the precuneus and temporo-parietal regions, and the middle frontal gyrus, consistently with the typical AD pattern of relative hypometabolism with respect to normalcy [35]. The temporal cortex and fusiform, supramarginal and angular gyri, with the highest extension in the left hemisphere, were significantly different in terms of glucose uptake in LMCI than EMCI, thereby capturing the most affected regions in patients progressing to dementia in a shorter term.

**Fig. 6:**
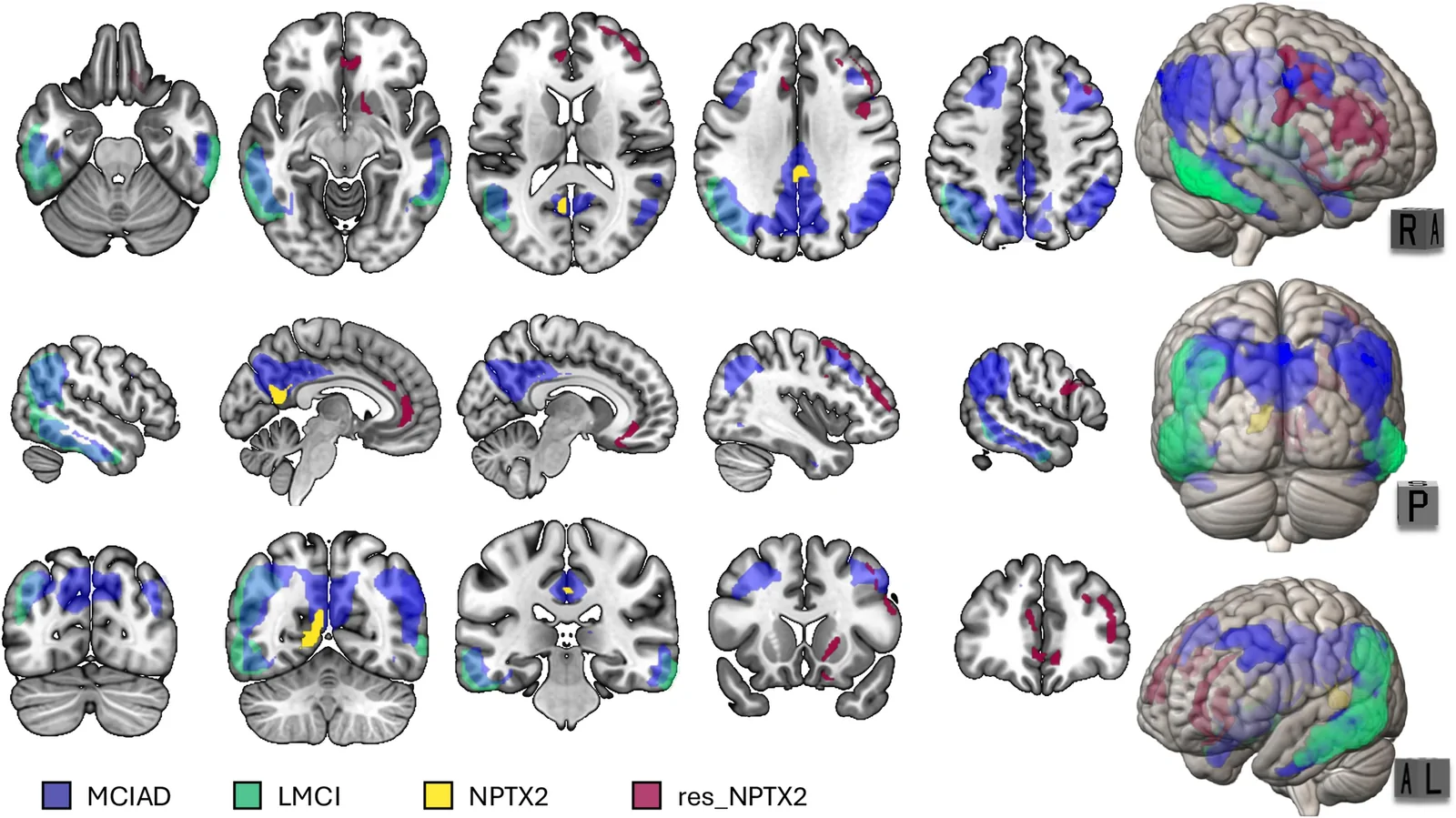
VBA analysis. The two-dimensional representation (axial, coronal and sagittal cuts) and three-dimensional rendering show the metabolic correlate of NPTX2 and Res_NPTX2 (in yellow and burgundy, respectively) superimposed on the MNI reference atlas (using MRIcroGL software, https://www.nitrc.org/projects/mricrogl). Their spatial relationship can be evaluated in relation to: (i) the MCI-AD VOIs (in blue), derived from the comparison between the MCI-AD and HC groups, and (ii) the relatively hypometabolic regions in LMCI than EMCI (in green) capturing the most affected (left) hemisphere in patients progressing to dementia in a shorter term. Abbreviations: HC, healthy controls; MCI-AD, Mild cognitive impairment due to Alzheimer’s disease; EMCI, early mild cognitive impairment; LMCI, late mild cognitive impairment; VOI, volumes of interest; NPTX2, neuronal pentraxin-2; Res_NPTX2, residual NPTX2 component (as defined in the text)

We observed a significant direct correlation of CSF NPTX2 with brain metabolism in posterior parietal regions, particularly in left precuneus/posterior cingulate cortex (PC/PCC) and lingual gyrus (NPTX2 VOI), consistently with our previous result in a smaller MCI-AD cohort [27]. After the incorporation of CSF Ng as a confounder in the regression model, this finding was no longer significant. Conversely, a direct correlation of Res_NPTX2 with brain metabolism was significant in fronto-striatal areas, namely caudate head, anterior cingulate cortex, and frontal gyri in the right hemisphere (Res_NPTX2 VOI),

Supplementary Table S6 details extension, coordinates, and corresponding Brodmann areas of the VOIs, which are displayed in Fig. 6.

## Discussion

Rates of cognitive deterioration vary among individuals with prodromal AD, while amyloid–tau core biomarkers alone do not fully account for this heterogeneity [2]. Synaptic alteration emerges as a plausible biological substrate of adaptive capacity and clinical resilience, which can be interpreted within a compensatory framework as the ability to maintain cognitive function or delay clinical progression despite AD pathology [43]. In this work, we aimed to operationalize this concept by integrating two CSF synaptic markers with complementary biological meaning: neurogranin (Ng), a postsynaptic protein widely interpreted as an index of AD-related synaptic injury [15, 16] and neuronal pentraxin-2 (NPTX2), a synaptic protein involved in activity-dependent synaptic remodeling and network homeostasis [19–26]. We show that extracting a residual CSF NPTX2 component (Res_NPTX2)—defined relative to age, CSF Ng and a core AD burden index —provides an operational measure of inter-individual differences in cognitive trajectories. Noteworthy, by combining pTau181 with the Aβ42/40 ratio, the AD burden index captured tau-related pathophysiology relative to amyloid abnormality while retaining the higher interpretability of Aβ42/40 over Aβ42 alone. This was used as a pragmatic adjustment variable to summarize core AD pathology within the residualization model, not as a novel diagnostic or staging biomarker.

Our first key observation is the confirmation that NPTX2 was the only CSF biomarker among those examined that significantly discriminated EMCI from LMCI, as defined by temporal proximity to dementia. By contrast, CSF Ng values were only nominally higher in EMCI than LMCI and did not remain significant after correction for multiple comparisons. This finding replicates our previous observation [27] in a partly overlapping, nearly doubled cohort and supports emerging evidence linking relatively preserved or increased NPTX2 to lower progression risk [28, 30], thereby challenging the notion of a monotonic CSF NPTX2 decline across the AD course paralleling cognitive deterioration [19].

The rationale to isolate a residual component of CSF NPTX2 arises from the observation that NPTX2 and Ng share part of their variance as CSF synaptic proteins, while also showing biologically and clinically divergent behavior [17, 25, 29]. Rather than viewing this partial covariation as a limitation, we explicitly addressed a different question, namely whether a component of NPTX2 exists that is not explained by synaptic injury alone but instead reflects adaptive synaptic remodeling and network capacity, interpretable within a compensatory framework. This conceptual separation aligns with recent large-scale proteomic work demonstrating that NPTX2-centered synaptic signatures capture clinically meaningful dimensions of cognitive resilience beyond amyloid and tau burden [30].

Such a premise is supported in our study by the correlation and median-split analyses of Ng and NPTX2, which revealed both shared variance and meaningful dissociation. Although the two markers show largely concordant behavior, the highest proportion of patients close to dementia conversion (LMCI) clustered in the low-NPTX2 group, irrespective of Ng levels. Conversely, higher CSF NPTX2 values—independent of Ng—were associated with a lower proportion of patients approaching clinical progression (LMCI). These patterns indicate that CSF NPTX2 carries clinically relevant information beyond synaptic injury. In such a scenario, the residual signal—rather than the raw concentration—becomes the relevant readout of a putativeNPTX2-related adaptive network capacity within the proposed compensatory framework. Conceptually, two individuals may show similar levels of synaptic injury (e.g., similar Ng) yet differ substantially in their putative capacity for compensatory network, captured here as higher versus lower NPTX2 relative to synaptic injury, AD pathological burden, and age. These covariates were explicitly included in the regression model to minimize confounding, and all predictors were z-transformed to address differences in scale. Multicollinearity among predictors was negligible, supporting their simultaneous inclusion in the residualization model. Importantly, Ng emerged as the only significant predictor of z(NPTX2), whereas age and AD burden did not, further supporting the interpretation that the shared variance between CSF NPTX2 and Ng reflects synaptic injury, while the residual captures an additional biological dimension.

Consistent with this interpretation, Res_NPTX2 was higher in EMCI than in LMCI and was significantly associated with better cognitive performance at BS and FU, as well as with a lower rate of cognitive decline as measured by ΔMMSE. Crucially, these associations remained robust after adjustment for MMSE BS and time of FU, indicating that Res_NPTX2 is not merely a proxy for baseline cognitive status or disease duration. In sensitivity analyses modelling synaptic markers directly, only NPTX2—but not Ng—was independently associated with longitudinal cognitive decline, supporting the view that NPTX2 captures a prognostically relevant dimension of synaptic function beyond injury-related processes that Res_NPTX2 can index.

Time-to-event analyses provided the strongest evidence for the prognostic value of Res_NPTX2, showing that higher levels were associated with a lower risk of progression to dementia and added incremental value beyond baseline cognitive status (i.e., MMSE BS). This pattern was further supported by tertile-based analyses of Res_NPTX2, where patients in the highest tertile showed significantly delayed progression to dementia compared with those in the middle or lowest tertiles, which had largely overlapping trajectories. The analyses consistently indicated an ordered low-to-high Res_NPTX2 gradient, with group differences predominantly driven by early progression events. Time-window analyses further refined the clinical interpretation. The protective effect of higher Res_NPTX2 was accentuated under two-year administrative censoring, whereas landmark analyses beyond two years showed no significant association. Mechanistically, neuronal pentraxins participate in the homeostatic adaptation of neuronal circuits to prolonged changes in activity, acting to rebalance excitation and inhibition [19, 24, 44]. NPTX2 gene expression is regulated by neuronal activity and synaptic plasticity demands, and its release promotes the stabilization of GluA4-containing AMPA receptors at excitatory synapses onto parvalbumin-positive interneurons, thereby supporting circuit-level control of excitability and rhythmicity [44, 45]. Beyond synaptic scaling, NPTX2 is implicated in neuroimmune regulation, through binding to complement component C1q and restraint of complement-mediated synapse elimination, providing a mechanistic link between synaptic compensation and protection from excessive microglial pruning [46]. Within this biological framework, synaptic compensation indexed by NPTX2—and more specifically by the Res_NPTX2 component—can be conceptualized as an intrinsically time-limited buffering mechanism, capable of delaying the clinical progression until compensatory homeostatic processes are progressively overwhelmed by AD pathology accumulation. However, the attenuation observed beyond 2 years may also reflect the smaller landmark sample, fewer late events, and increasing censoring, warranting caution in interpreting this temporal pattern.

A second contribution of this study is exploring an interpretable stratification framework. We emphasize that this exploratory approach is not intended to define a diagnostic biomarker for clinical practice, but rather to propose a model for profiling patients according to progression risk and deepening biological interpretation. By anchoring CSF NPTX2 values to a non-demented control (OND) distribution, we defined clinically interpretable NPTX2 bands referenced to an external comparator. Crucially, cross-classifying NPTX2_OND_ bands with Res_NPTX2 tertiles further sharpened risk stratification into putative compensation-related phenotypes. Notably, no patients with low NPTX2_OND_ exhibited high Res_NPTX2 values. More importantly, none of the 24 patients characterized by both high NPTX2_OND_ and high Res_NPTX2 progressed from MCI to dementia within two years, highlighting a subgroup with particularly low short-term risk. This two-dimensional architecture provides a concrete and intuitive way to convey that synaptic injury and a statistically isolated, putative compensatory capacity represent distinct biological dimensions that, when considered jointly, may offer a more nuanced characterization of synaptopathy in AD.

This residual-based framing is not merely a statistical construct but aligns with a plausible biological model. Preserved or relatively increased CSF NPTX2 in early prodromal AD may reflect.

an adaptive synaptic response compatible with compensation-related mechanisms, consistent with studies showing that NPTX2 behaves differently from classical synaptic injury markers and tracks dimensions of cognitive resilience beyond amyloid and tau burden [17, 28–30]. More broadly, recent evidence on other neuronal pentraxins further supports the relevance of this synaptic pathway in AD: CSF NPTX1 and NPTXR progressively decrease with disease severity, correlate with cortical thinning and cognitive impairment, and predict subsequent dementia progression, reinforcing the view that the neuronal pentraxin family captures clinically meaningful aspects of synaptic functioning across the AD continuum [47].

Converging evidence identified right anterior frontal networks as primary substrates of cognitive reserve and resilience in AD [32, 48–50]. In contrast, left-sided temporo-parietal regions are among the earliest and most vulnerable metabolic targets of the disease [51]. Consistently, LMCI showed lower glucose uptake than EMCI in temporo-parietal cortices, with the greatest extension in the left hemisphere. Noteworthy, CSF NPTX2 was directly associated with metabolism in left posterior parietal regions, particularly the PC/PCC, but this association was no longer significant after adjustment for Ng. This suggests that the posterior NPTX2–FDG signal substantially overlaps with Ng-related synaptic injury burden, in line with previous evidence mapping Ng-specific metabolic correlates onto the PC/PCC in MCI-AD [32].

By contrast, Res_NPTX2 was associated with metabolism in right-lateralized fronto-striatal regions, partly outside the canonical posterior AD hypometabolic pattern. This hemispheric and topographical dissociation is biologically informative. The left-weighted posterior pattern identifies the metabolically most vulnerable territory in patients closer to dementia conversion, whereas the right fronto-striatal pattern points to a distinct system more consistent with compensatory recruitment. One possibility is that this pattern indexes preserved executive-control or salience-related circuitry that temporarily buffers clinical expression of pathology. Another is that it reflects a more general marker of disease stage or network efficiency. This interpretation aligns with reserve and resilience models in AD, whereby right frontal regions – especially the right dorsolateral prefrontal cortex - support executive control and sustained attention when networks become progressively disconnected [49, 50]. In late converters, preserved fronto-striatal efficiency may partially shield cognitive performance from increasing pathological load [52, 53]. Crucially, this compensatory mechanism is time-dependent. Changes in synaptic stability and excitation–inhibition balance may drive dynamic connectivity alterations in AD, from early relative hyperconnectivity to later hypoconnectivity as neurodegeneration progresses [7]. Within this framework, Res_NPTX2 may index a time-limited phase in which right fronto-striatal recruitment remains clinically effective before compensatory mechanisms are progressively exhausted.

A predictable concern is that residualization may appear unnecessarily complex, especially since raw CSF NPTX2 can show comparable discrimination between EMCI and LMCI, and residual-based metrics are inherently cohort-dependent. We therefore do not interpret Res_NPTX2 as a universally superior predictor replacing raw NPTX2. Rather, in line with the mechanistic nature of the study, the added value of Res_NPTX2 lies in its mechanistic interpretability. By explicitly capturing the component of NPTX2 that is independent of synaptic injury and more strongly associated with delayed progression, this approach provides a biologically interpretable link between molecular synaptic mechanisms and resilience/compensatory mechanisms. Because the cohort was intentionally restricted to biologically defined MCI-AD patients, pTau181 and Aβ42/40 were not considered competing predictors but rather markers to be incorporated into the AD burden adjustment. Therefore, the aim of the study was not to outperform core AD biomarkers. In the context of recent NIA–AA criteria, which emphasize tau-based metrics for biological staging [2], our findings provide a complementary framework to capture inter-individual heterogeneity in clinical trajectories among patients with comparable AD burden. This is in line with emerging models of healthy cognitive aging proposing that such variability may depend on the integrity of core network-regulatory mechanisms, particularly synaptic plasticity, excitation/inhibition balance, and large-scale network organization [54].

This study has several strengths, including a clinically well-characterized cohort with biomarker-supported MCI-AD diagnosis aligned with current criteria [1, 2], robust statistical methods with sensitivity analyses and control for multiple comparisons. Anchoring NPTX2 bands to an external reference group further limited circularity and enabled a two-layer compensation framework that improved biological and clinical interpretability. An additional strength is the availability of [^18^F]FDG PET data. This translational imaging layer enriches the study by providing anatomically grounded support for the proposed mechanistic interpretation, particularly the distinction between posterior vulnerability-related signals and a fronto-striatal, compensation-related network signature. In this sense, the imaging findings reinforce the biological plausibility of Res_NPTX2 as a marker of network-level adaptive capacity. Another point relates to the operational definition of EMCI and LMCI. Consistently with our previous research [27, 32], these categories were identified a priori to reflect proximity to dementia conversion rather than disease duration, which is difficult to estimate reliably in MCI because symptom onset is gradual and lacks a clear index timepoint. Alternative staging approaches, including cognitive-score-based classifications, may capture different aspects of disease heterogeneity [55, 56]. Nevertheless, the progression-based framework consistently identified NPTX2-related differences associated with short-term clinical progression.

Limitations include the single-center retrospective design and the need for validation in larger cohorts spanning from cognitively unimpaired to demented individuals. Importantly, the compensatory interpretation remains inferential. The present observational design can identify biomarker and metabolic patterns compatible with adaptive network capacity but cannot demonstrate active compensation or causal recruitment of compensatory circuits. Furthermore, residual-based constructs are inherently model-dependent and cognitive outcomes relied on MMSE and clinical conversion, which may have limited sensitivity to subtle change. Finally, although we adjusted for age and AD burden, other modifiers—such as cognitive reserve [43], genetic factors, and co-pathologies—were not explicitly modeled and may refine interpretation in larger datasets. As expected for a conventional reserve proxy, education was associated with MMSE BS, but not with longitudinal cognitive measures or CSF biomarkers. This supports the possibility that Res_NPTX2 captures a more proximal biological dimension of synaptic/network adaptive capacity rather than education-related reserve alone. No competing events, including non-AD dementia or death before AD dementia progression, were observed. However, symptomatic treatment exposure was not systematically accounted for in the prognostic analyses and may represent a source of residual confounding. Also, the OND reference group was clinically heterogeneous and included few strictly healthy individuals; therefore, the OND-derived NPTX2 bands require validation in larger and more homogeneous cognitively unimpaired cohorts. Overall, the present findings should be regarded as hypothesis-generating, and replication in larger, independent biomarker-defined AD cohorts using the same analytical framework will be necessary to establish the generalizability of this cohort-calibrated residual metric.

In conclusion, in biomarker-confirmed MCI-AD, CSF NPTX2 carries prognostic information associated with better cognition, lower short-term progression risk, and a right fronto-striatal metabolic signature supporting a biologically plausible adaptive network signal. Within a compensatory framework, the proposed residualization approach provides a statistical strategy to disentangle a putative NPTX2-related adaptive-capacity component from variance shared with synaptic injury and AD pathological burden. CSF NPTX2 therefore emerges as a promising complement to core AD biomarkers, providing biologically interpretable information on short-term progression risk and clinically relevant heterogeneity in early AD.

## Supplementary Information

Supplementary Material 1.

## Data Availability

Data are available on reasonable request from the corresponding author.
