# Supplementary material for: A CSF NPTX2-based residual metric captures prognostic heterogeneity within a proposed compensatory framework in prodromal Alzheimer’s disease
Source: Alzheimers Res Ther. 2026 Aug 11;18:244. doi: 10.1186/s13195-026-02160-y (PMC13746400; doi:10.1186/s13195-026-02160-y)
Supplement: Supplementary file 1 [file 13195_2026_2160_MOESM1_ESM.zip › Supplementary Figures__Res_NPTX2__rev.docx]

**Supplementary Figure S1. Distribution of Ng–NPTX2 quadrants differs between EMCI and LMCI.** Bar plot showing the counts of participants in each Ng x NPTX2 median-split quadrant within EMCI and LMCI. Group differences in quadrant distribution were tested with a chi-square test (χ²(3)=12.31, p=0.006), indicating a non-random association between progression group and Ng–NPTX2 quadrant membership.

**Supplementary Figure S2. LMCI rate by CSF NPTX2_OND_ bands and Res_NPTX2 tertiles**

Heatmap showing the proportion of short-term converters (LMCI, progression to AD dementia within 2 years from baseline) across the 3×3 cross-classification of (i) OND-anchored CSF NPTX2 (NPTX2_OND_) bands (rows with number of participants (n) in each: Low/Mid/High defined using the OND distribution at ±0.5 SD around the OND mean; Low ≤569.5 pg/mL, High ≥1096.3 pg/mL; Mid otherwise) and (ii) Res_NPTX2 tertiles (columns: Low/Mid/High; derived within the MCI-AD cohort). Each cell reports the LMCI rate (%) with events/total in parentheses. The crossed cell indicates no participants. Color scale represents LMCI rate (0–100%).

**Supplementary Figure S3.** Receiver operating characteristic (ROC) curves for CSF NPTX2 (green), Res_NPTX2 (purple), and CSF neurogranin (Ng, pink). CSF NPTX2 (AUC=0.73) and Res_NPTX2 (AUC=0.71) showed similar discrimination of LMCI versus EMCI, whereas Ng showed lower discrimination (AUC=0.63). The dashed diagonal line indicates chance-level performance (AUC=0.50). No formal comparison between ROC curves was performed.
