# Supplementary material for: A CSF NPTX2-based residual metric captures prognostic heterogeneity within a proposed compensatory framework in prodromal Alzheimer’s disease
Source: Alzheimers Res Ther. 2026 Aug 11;18:244. doi: 10.1186/s13195-026-02160-y (PMC13746400; doi:10.1186/s13195-026-02160-y)
Supplement: Supplementary file 1 [file 13195_2026_2160_MOESM1_ESM.zip › Supplementary materials.docx]

**Supplementary methods**

**CSF collection and pre-processing.** Cerebrospinal fluid (CSF) samples (6–8 mL) were obtained by lumbar puncture at the L3–L4 or L4–L5 interspace in the early morning, according to standardized procedures [1]. Samples were collected into sterile polypropylene tubes and centrifuged at 4000 g for 10 minutes at 4°C, then aliquoted into polypropylene tubes and stored at −80°C until analysis to ensure long-term protein stability.

**FDG-PET protocol and image pre-processing.**

Baseline [18F]FDG-PET scans were acquired in accordance with European Association of Nuclear Medicine (EANM) guidelines [2, 3]. Participants fasted for at least 6 hours and had blood glucose levels below 7.8 mmol/L at the time of scanning. Prior to tracer injection, subjects rested for 10 minutes in a quiet, dimly lit room with eyes open and minimal sensory stimulation. A dose of 185–250 MBq of [^18^F]FDG was administered intravenously, and image acquisition started approximately 45 minutes later, with a scan duration of 15 minutes, using a Siemens Biograph 16 PET/CT system. Images were reconstructed using an ordered subset expectation maximization algorithm, following standard clinical protocols, with CT-based attenuation correction.

Pre-processing was performed using MATLAB and Statistical Parametric Mapping (SPM12; Wellcome Trust Centre for Neuroimaging, London, UK). Images were spatially normalized to an FDG-PET template for neurodegenerative diseases in MNI space [4], resampled to 2 × 2 × 2 mm³ voxels, and smoothed with a 10-mm full-width at half-maximum isotropic Gaussian kernel. All images were visually inspected for quality control by two experienced readers (FM and SR) to ensure accuracy of preprocessing.

**Supplementary references**

Arbizu J, Morbelli S, Minoshima S, et al (2025) SNMMI Procedure Standard/EANM Practice Guideline for Brain [18F]FDG PET Imaging, Version 2.0. J Nucl Med 66:S45–S60. https://doi.org/10.2967/JNUMED.124.268754

Della Rosa PA, Cerami C, Gallivanone F, et al (2014) A standardized [18 F]-FDG-PET template for spatial normalization in statistical parametric mapping of dementia. Neuroinformatics 12:575–593

Guedj E, Varrone A, Boellaard R, et al (2022) EANM procedure guidelines for brain PET imaging using [18F]FDG, version 3. Eur J Nucl Med Mol Imaging 49:632–651. <https://doi.org/10.1007/s00259-021-05603-w>

Teunissen CE, Petzold A, Bennett JL, et al (2009) A consensus protocol for the standardization of cerebrospinal fluid collection and biobanking. Neurology 73:1914–1922. https://doi.org/10.1212/WNL.0b013e3181c47cc2
