# Supplementary material for: A CSF NPTX2-based residual metric captures prognostic heterogeneity within a proposed compensatory framework in prodromal Alzheimer’s disease
Source: Alzheimers Res Ther. 2026 Aug 11;18:244. doi: 10.1186/s13195-026-02160-y (PMC13746400; doi:10.1186/s13195-026-02160-y)
Supplement: Supplementary file 1 [file 13195_2026_2160_MOESM1_ESM.zip › SupplTable_S1_correlations.docx]

**Table 1s. Age-adjusted Spearman’s correlations**

|  |  | Aβ42/Aβ40 | p-Tau181 | t-Tau | NPTX2 | Ng | Edu | MMSE BS |
| --- | --- | --- | --- | --- | --- | --- | --- | --- |
| p-Tau181 | Spearman's rho | -0.29 |  |  |  |  |  |  |
|  | *p* value | **0.003** |  |  |  |  |  |  |
| t-Tau | Spearman's rho | -0.22 | 0.95 |  |  |  |  |  |
|  | *p* value | **0.024** | **< 0.001** |  |  |  |  |  |
| NPTX2 | Spearman's rho | -0.30 | 0.32 | 0.37 |  |  |  |  |
|  | *p* value | **0.002** | **< 0.001** | **< 0.001** |  |  |  |  |
| Ng | Spearman's rho | -0.31 | 0.65 | 0.68 | 0.67 |  |  |  |
|  | *p* value | **0.001** | **<0.001** | **<0.001** | **<0.001** |  |  |  |
| EDU | Spearman's rho  *p* value | 0.11 | -0.10 | -0.09 | -0.07 | -0.12 |  |  |
|  |  | 0.276 | 0.32 | 0.380 | 0.503 | 0.215 |  |  |
| MMSE BS | Spearman's rho  *p* value | -0.09  0.374 | 0.03  0.76 | 0.04  0.671 | 0.19  **0.050** | 0.05  0.580 | 0.27  **0.006** |  |
| MMSE FU | Spearman's rho  *p* value | -0.08  0.441 | -0.29  **0.003** | -0.23  **0.018** | 0.38  **<0.001** | 0.10  0.310 | 0.17  0.095 | 0.38  **<0.001** |

Statistical significant p-values are in **bold**.

Aβ42/Aβ40 ratio amyloid Aβ1-42 normalized for amyloid Aβ1-40, pTau181 Tau phosphorylated at threonine 181, t-Tau total Tau, Cerebrospinal fluid, NPTX2 Neuronal Pentraxin 2, Ng Neurogranin, Edu, education, MMSE Mini Mental State Examination, BS Baseline, FU Follow-up
