# Supplementary material for: A CSF NPTX2-based residual metric captures prognostic heterogeneity within a proposed compensatory framework in prodromal Alzheimer’s disease
Source: Alzheimers Res Ther. 2026 Aug 11;18:244. doi: 10.1186/s13195-026-02160-y (PMC13746400; doi:10.1186/s13195-026-02160-y)
Supplement: Supplementary file 1 [file 13195_2026_2160_MOESM1_ESM.zip › SupplTable_S2_zNPTX2_zNg_DeltaMMSE.docx]

**Supplementary Table S2. Independent and joint contributions of z(NPTX2) and z(Ng) to MMSE decline over the observation period (ΔMMSE)**

| **Model** | **Predictor** | **β**  **(95% CI)** | **partial R²** | **Model R²** | **p-values** |
| --- | --- | --- | --- | --- | --- |
| **ΔMMSE ~ z(NPTX2)**  **+ MMSE BS + FU** | z(NPTX2) | -1.55  (-2.50 to -0.61) | 0.097 | 0.24 | **0.001** |
|  | MMSE BS | 0.06  (-0.31 to 0.43) | 0.001 |  | 0.759 |
|  | FU (years) | 1.03  (0.58 to 1.48) | 0.169 |  | **<0.001** |
| **ΔMMSE ~ z(Ng)**  **+ MMSE BS + FU** | z(Ng) | -0.43  (-1.39 to 0.53) | 0.008 | 0.17 | 0.380 |
|  | MMSE BS | -0.08  (-0.46 to 0.30) | 0.002 |  | 0.672 |
|  | FU (years) | 1.05  (0.58 to 1.52) | 0.162 |  | **<0.001** |
| **ΔMMSE ~ z(NPTX2) + z(Ng)**  **+ MMSE BS + FU** | z(NPTX2) | -1.95  (-3.10 to -0.80) | 0.102 | 0.25 | **0.001** |
|  | z(Ng) | 0.66  (-0.46 to 1.78) | 0.014 |  | 0.243 |
|  | MMSE BS | 0.08  (-0.29 to 0.45) | 0.002 |  | 0.665 |
|  | FU (years) | 1.02  (0.57 to 1.47) | 0.169 |  | **<0.001** |

Outcome is ΔMMSE = MMSE BS – MMSE FU (positive values indicate cognitive decline).

Models are ordinary least-squares regressions including baseline MMSE and FU duration (years). z(NPTX2) and z(Ng) are baseline cohort-standardised values (1 unit = 1 SD).

Significant p-values are in **bold**.
