# Supplementary material for: A CSF NPTX2-based residual metric captures prognostic heterogeneity within a proposed compensatory framework in prodromal Alzheimer’s disease
Source: Alzheimers Res Ther. 2026 Aug 11;18:244. doi: 10.1186/s13195-026-02160-y (PMC13746400; doi:10.1186/s13195-026-02160-y)
Supplement: Supplementary file 1 [file 13195_2026_2160_MOESM1_ESM.zip › SupplTable_S3_Cox models.docx]

**Supplementary Table S3. Cox regression models for time-to-progression to AD dementia**

| **Model** | **Predictors** | **β (SE)** | **HR** | **95% CI** | **AIC** | **p-value** |  |
| --- | --- | --- | --- | --- | --- | --- | --- |
| Main | Res_NPTX2 | −0.46 (0.17) | 0.63 | 0.45–0.88 | 384.7 | **0.007** |  |
| Sensitivity 1 | MMSE BS | −0.25 (0.05) | 0.78 | 0.71–0.87 | 372.5 | **<0.001** |  |
| Sensitivity 2 | MMSE BS | −0.22 (0.05) | 0.80 | 0.72–0.89 | 368.8 | **<0.001** |  |
|  | Res_NPTX2 | −0.45 (0.20) | 0.64 | 0.43–0.93 |  | **0.025** |  |

Notes: N=104 (events=55; censored=49). Ties=6, handled with the Exact method.

HR indicates hazard ratio per 1-unit increase in the corresponding predictor. 95% CIs are profile-likelihood CIs. Model fit is summarized by the Akaike information criterion (AIC) (lower values indicate better relative support). Significant p-values are in **bold**.
