# Supplementary material for: A CSF NPTX2-based residual metric captures prognostic heterogeneity within a proposed compensatory framework in prodromal Alzheimer’s disease
Source: Alzheimers Res Ther. 2026 Aug 11;18:244. doi: 10.1186/s13195-026-02160-y (PMC13746400; doi:10.1186/s13195-026-02160-y)
Supplement: Supplementary file 1 [file 13195_2026_2160_MOESM1_ESM.zip › SupplTable_S4_Table_KM_Cox_Res_NPTX2_rev.docx]

**Supplementary Table S4. Summary of Kaplan–Meier (KM) and Cox analyses across Res_NPTX2 tertiles**

| **Observation period** | **Model / Test** | **Contrast** |  | **95% CI** | **p-value** |
| --- | --- | --- | --- | --- | --- |
| **Full follow-up** | KM (3 groups) | Log-rank (Mantel–Cox) | χ²=9.43  (df=2) |  | **0.009** |
|  |  | Log-rank test for trend | χ²=7.86  (df=1) |  | **0.005** |
|  |  | Gehan–Breslow-Wilcoxon^1^ | χ²=9.02  df=2) |  | **0.011** |
|  | KM pairwise (log-rank)^2^ | Mid vs Low |  |  | p=0.965  p_adj=0.965 |
|  |  | High vs Low |  |  | **p=0.003**  **p_adj=0.006** |
|  |  | High vs Mid |  |  | **p=0.002**  **p_adj=0.006** |
|  | Cox (dummy; Low ref)^3^ | Mid vs Low | HR=1.04 | 0.57–1.90 | 0.897 |
|  |  | High vs Low | HR=0.35 | 0.16–0.74 | **0.006** |
|  |  |  |  |  |  |
| **≤2y administrative censoring^4^** | Cox (trend; per tertile) | per +1 tertile | HR=0.50 | 0.30–0.82 | **0.006** |
|  | Cox (dummy; Low ref) ^3^ | Mid vs Low | HR=0.74 | 0.33–1.63 | 0.452 |
|  | Cox (dummy; Low ref) ^3^ | High vs Low | HR=0.18 | 0.05–0.64 | **0.008** |
| **>2y landmark^5^** | Cox (trend; per tertile) | per +1 tertile | HR=0.89 | 0.55–1.43 | 0.624 |

**^1^** Gehan–Breslow–Wilcoxon test gives greater weight to early events.

**^2^** Pairwise p-values are from two-group Mantel–Cox (log-rank) tests; p_adj values are Holm–Šidák–adjusted for 3 pairwise comparisons.

**^3^** Cox dummy model uses tertiles encoded as indicator variables with Low as the reference category (HRs for Mid vs Low and High vs Low).

**^4^** Administrative censoring sensitivity: follow-up truncated at 2 years, event only for conversions within 2 years (event=1 and time≤2); all others censored at 2 years

**^5^** 2-year landmark analysis restricted to participants event-free at 2 years (time>2), with time re-indexed from the landmark.

Significant p-values in **bold**.

**Abbreviations:** KM, Kaplan–Meier; Cox, Cox proportional-hazards regression; HR, hazard ratio; CI, confidence interval; FU, follow-up; df, degrees of freedom; χ², chi-square statistic; p_adj, multiplicity-adjusted p value (Holm–Šidák); Low/Mid/High, tertiles of Res_NPTX2; time_2y, administratively censored follow-up time truncated at 2 years; event_2y, event indicator within 2 years; time_post2, follow-up time re-indexed from the 2-year landmark.
