# Supplementary material for: A CSF NPTX2-based residual metric captures prognostic heterogeneity within a proposed compensatory framework in prodromal Alzheimer’s disease
Source: Alzheimers Res Ther. 2026 Aug 11;18:244. doi: 10.1186/s13195-026-02160-y (PMC13746400; doi:10.1186/s13195-026-02160-y)
Supplement: Supplementary file 1 [file 13195_2026_2160_MOESM1_ESM.zip › SupplTable_S5_compensation_phenotypes.docx]

**Supplementary Table S5. Two-layer compensation phenotypes (OND-anchored NPTX2 bands + Res_NPTX2 tertiles) and progression outcomes.**

| **Phenotype** | **Definition** | **n** | **LMCI**  **n (%)** | **EMCI**  **n** | **Progression**  **n (%)** |
| --- | --- | --- | --- | --- | --- |
| Compensation-preserved | High NPTX2_OND_ band + High Res_NPTX2 tertile | 24 | 0 (0.0) | 24 | 2 (8.3) |
| Intermediate | All other combinations | 65 | 22 (33.8) | 43 | 42 (64.6) |
| Compensation-failure | Low NPTX2OND band + Low Res_NPTX2 tertile | 15 | 8 (53.3) | 7 | 11 (73.3) |
| **Total** |  | **104** | **30 (28.8)** | **74** | **55 (52.9)** |

**Notes:** LMCI indicates progression to AD dementia within 2 years from baseline. 'Progression ' indicates conversion during the whole available follow-up.

Association with LMCI across the three phenotypes: χ²(2)=14.90, p=0.0006; Cramer's V=0.38. Association with overall progression (any time): χ²(2)=25.22, p<0.001; Cramer's V=0.49.
