# Supplementary material for: A CSF NPTX2-based residual metric captures prognostic heterogeneity within a proposed compensatory framework in prodromal Alzheimer’s disease
Source: Alzheimers Res Ther. 2026 Aug 11;18:244. doi: 10.1186/s13195-026-02160-y (PMC13746400; doi:10.1186/s13195-026-02160-y)
Supplement: Supplementary file 1 [file 13195_2026_2160_MOESM1_ESM.zip › SupplTable_S6_FDG clusters.docx]

**Supplementary Table S6. Significant brain areas resulting from the VBA of [^18^F]FDG PET scans**

| **CTR vs MCI AD** | | | | | | | | | | | | |
| --- | --- | --- | --- | --- | --- | --- | --- | --- | --- | --- | --- | --- |
| **VOI**  **(Cluster extent)** | **Peak level p-value** | | | **Cluster peaks coordinates** | | | | | | | **Cortical region (gyrus)** | **BA** |
|  |  | | | **x** | | **y** | | | **z** | |  |  |
| MCI-AD VOI1 | p < 0.001 | | | -7.08 | | -63.97 | | | 25.12 | | Left precuneus | 31 |
| (10176) |  | | | -36.82 | | -68.42 | | | 33.21 | | Left angular gyrus |  |
|  |  | | | -1.56 | | -49.62 | | | 31.98 | | Left precuneus | 31 |
|  |  | | | 0.28 | | -46.08 | | | 34.15 | | Left precuneus | 31 |
|  |  | | | -1.58 | | -40.65 | | | 36.43 | | Left cingulate gyrus | 31 |
|  |  | | | 5.88 | | -62.18 | | | 25.51 | | Right precuneus | 31 |
|  |  | | | -47.67 | | -59.16 | | | 15.88 | | Left middle temporal gyrus | 21 |
|  |  | | | -47.79 | | -52.76 | | | 27.3 | | Left supramarginal gyrus |  |
|  |  | | | -36.06 | | -31.69 | | | -15.55 | | Left parahippocampal gyrus | 30 |
|  |  | | | -37.83 | | -22.02 | | | -18.27 | | Left parahippocampal gyrus | 30 |
|  |  | | | 17.3 | | -48.97 | | | 5.34 | | Right parahippocampal gyrus | 30 |
|  |  | | | -16.43 | | -57.34 | | | 34.6 | | Left precuneus | 31 |
|  |  | | | -37.92 | | -37.27 | | | -16.11 | | Left fusiform gyrus | 20 |
| MCI-AD VOI2 |  | | | 46.58 | | -58.84 | | | 28.32 | | Right superior temporal gyrus | 39 |
| (4097) |  | | | 50.87 | | -39.94 | | | -11.26 | | Right fusiform gyrus | 37 |
|  |  | | | 48.42 | | -46.15 | | | 33.15 | | Right supramarginal gyrus |  |
|  |  | | | 44.59 | | -52.59 | | | 41.49 | | Right inferior parietal lobule |  |
|  |  | | | 41.96 | | -2.58 | | | -27.69 | | Right middle temporal gyrus | 21 |
|  |  | | | 39.81 | | -37.67 | | | -14.83 | | Right parahippocampal gyrus | 30 |
|  |  | | | 39.78 | | -41.57 | | | -13.4 | | Right fusiform gyrus | 20 |
|  |  | | | 42.7 | | -76.63 | | | 37.38 | | Right precuneus | 31 |
|  |  | | | 44.59 | | -76.29 | | | 33.84 | | Right angular gyrus |  |
| MCI-AD VOI3 |  | | | 31.76 | | 5.06 | | | 48.53 | | Right middle frontal gyrus | 6 |
| (987) |  | | | 33.86 | | 23.39 | | | 34.09 | | Right middle frontal gyrus | 9 |
|  |  | | | 46.6 | | 8.88 | | | 47.35 | | Right middle frontal gyrus | 6 |
|  |  | | | 50.33 | | 5.49 | | | 43.48 | | Right middle frontal gyrus | 6 |
|  |  | | | 44.7 | | 10.41 | | | 51.06 | | Right middle frontal gyrus | 6 |
|  |  | | | 18.81 | | -0.29 | | | 46.01 | | Right cingulate gyrus | 31 |
|  |  | | | -7.08 | | -63.97 | | | 25.12 | | Left precuneus | 31 |
|  |  | | | -36.82 | | -68.42 | | | 33.21 | | Left angular gyrus |  |
|  |  | | | 46.58 | | -58.84 | | | 28.32 | | Right superior temporal gyrus | 39 |
|  |  | | | 50.87 | | -39.94 | | | -11.26 | | Right fusiform gyrus | 37 |
| MCI-AD VOI4 |  | | | 31.76 | | 5.06 | | | 48.53 | | Right middle frontal gyrus | 6 |
| (1186) |  | | | 33.86 | | 23.39 | | | 34.09 | | Right middle frontal gyrus | 9 |
|  |  | | | 46.6 | | 8.88 | | | 47.35 | | Right middle frontal gyrus | 6 |
|  |  | | | -29.28 | | 11.14 | | | 46.28 | | Left middle frontal gyrus | 6 |
|  |  | | | -36.59 | | 13.74 | | | 39.19 | | Left middle frontal gyrus | 6 |
| **EMCI vs LMCI** | | | | | | | | | | | | |
| **Cluster extent** | **Cluster Significance** | | | **Cluster peaks coordinates** | | | | | | | **Cortical region (gyrus)** | **BA** |
|  |  | | | **x** | | **y** | | | **z** | |  |  |
| Cluster 1 | p < 0.001 | | | -56.54 | | -43.46 | | | -9.81 | | Left middle temporal gyrus | 21 |
| (6268) |  | | | -51.49 | | -58.15 | | | 24.92 | | Left superior temporal gyrus | 39 |
|  |  | | | -44.19 | | -64.48 | | | 31.65 | | Left angular gyrus |  |
|  |  | | | -45.99 | | -64.12 | | | 28.05 | | Left middle temporal gyrus | 21 |
|  |  | | | -54.5 | | -25.66 | | | -18.89 | | Left inferior temporal gyrus | 20 |
|  |  | | | -46.06 | | -60.92 | | | 33.76 | | Left angular gyrus |  |
|  |  | | | -54.47 | | -21.76 | | | -20.33 | | Left inferior temporal gyrus | 20 |
|  |  | | | -41.61 | | -40.8 | | | -18.31 | | Left fusiform gyrus | 37 |
|  |  | | | -47.22 | | -44.85 | | | -15.18 | | Left fusiform gyrus | 37 |
|  |  | | | -51.48 | | -47.15 | | | 27.77 | | Left supramarginal gyrus |  |
|  |  | | | | -46.89 | -0.43 | | | -27.19 | Left middle temporal gyrus | | 21 |
| Cluster 2  (1319) |  | | | | 60.17 | -37.78 | | | -14.5 | Right middle temporal gyrus | | 21 |
|  |  | | | | 60.25 | -24.39 | | | -16.83 | Right inferior temporal gyrus | | 20 |
|  |  | | | | 53.01 | -6.71 | | | -24.29 | Right fusiform gyrus | | 20 |
|  |  | | | | 47.14 | -51.1 | | | -12.38 | Right fusiform gyrus | | 37 |
|  |  | | | | 54.39 | -55.91 | | | -1.9 | Right middle temporal gyrus | | 21 |
| Cluster 3 |  | | | | 50.18 | -54.14 | | | 37.83 | Right supramarginal gyrus | |  |
| (1333) |  | | | | 48.47 | -62.22 | | | 24.43 | Right middle temporal gyrus | | 21 |
|  |  | | | | 44.67 | -66.63 | | | 31.15 | Right angular gyrus | |  |
| **CSF NPTX2** | | | | | | | | | | | | |
| **Cluster extent** | | **Cluster Significance** | | | **Cluster peaks coordinates** | | | | | **Cortical region (gyrus)** | | **BA** |
|  | |  | | | **x** | **y** | | | **z** |  | |  |
| NPTX2-VOI | | p < 0.001 | | | -3.31 | -37.91 | | | 27.66 | Left cingulate gyrus | | 31 |
| (345) | |  | | | -15.99 | -57.76 | | | 0.34 | Left lingual gyrus | | 19 |
|  | |  | | | -1.51 | -29.12 | | | 33.92 | Left cingulate gyrus | | 31 |
|  | |  | | | -12.28 | -54.05 | | | 0.75 | Left lingual gyrus | | 19 |
|  | |  | | | -8.65 | -54.59 | | | 6.17 | Left posterior cingulate | | 30 |
|  | |  | | | -5.12 | -48.55 | | | 21.21 | Left posterior cingulate | | 30 |
|  | |  | | | -5.08 | -51.93 | | | 17.29 | Left posterior cingulate | | 30 |
|  | |  | | | -10.76 | -65.64 | | | 23.1 | Left precuneus | | 31 |
| **Res_NPTX2** | | | | | | | | | | | | |
| **Cluster extent** | | | **Cluster Significance** | | **Cluster peaks coordinates** | | | | | **Cortical region (gyrus)** | | **BA** |
|  | | |  | | **x** | | **y** | **z** | |  | |  |
| Cluster 1 | | | p < 0.002 | | -8.52 | | 32.28 | 21.6 | | Left anterior cingulate | |  |
| (605) | | |  | | 11.99 | | 11.38 | 3.76 | | Right caudate | |  |
|  | | |  | | -8.44 | | 36.53 | 16.6 | | Left anterior cingulate | |  |
|  | | |  | | -6.79 | | 22.26 | 27.89 | | Left cingulate gyrus | | 32 |
|  | | |  | | 10.19 | | 11.74 | 0.16 | | Right caudate | |  |
|  | | |  | | 12.23 | | 14.99 | -13.91 | | Right subcallosal gyrus | |  |
|  | | |  | | -8.73 | | 16.15 | 32.69 | | Left cingulate gyrus | | 32 |
|  | | |  | | 12.27 | | 18.89 | -15.35 | | Right medial frontal gyrus | | 25 |
|  | | |  | | 8.44 | | 34.46 | -1.32 | | Right anterior cingulate | | 24 |
|  | | |  | | 4.77 | | 27.37 | -5.66 | | Right anterior cingulate | | 24 |
|  | | |  | | 8.41 | | 12.27 | -5.23 | | Right caudate | |  |
|  | | |  | | 8.62 | | 32.12 | -15.96 | | Right medial frontal gyrus | | 9 |
| Cluster 2 | | |  | | 35.98 | | 47.3 | 20.18 | | Right middle frontal gyrus | | 6 |
| (875) | | |  | | 35.85 | | 40.84 | 28.57 | | Right middle frontal gyrus | | 6 |
|  | | |  | | 37.51 | | 1.01 | 32.03 | | Right inferior frontal gyrus | | 46 |
|  | | |  | | 37.18 | | 0.43 | 57.2 | | Right middle frontal gyrus | | 6 |
|  | | |  | | 43.01 | | 18.91 | 41.03 | | Right middle frontal gyrus | | 6 |
|  | | |  | | 19.3 | | 54.49 | 24.18 | | Right superior frontal gyrus | | 10 |
|  | | |  | | 22.99 | | 52.61 | 24.06 | | Right superior frontal gyrus | | 10 |
|  | | |  | | 21.09 | | 50.41 | 27.43 | | Right superior frontal gyrus | | 10 |
|  | | |  | | 50.55 | | 8.92 | 27.6 | | Right inferior frontal gyrus | | 46 |
|  | | |  | | 48.66 | | 10.44 | 31.31 | | Right middle frontal gyrus | | 6 |
|  | | |  | | 31.76 | | 16.06 | 51.38 | | Right superior frontal gyrus | | 8 |
|  | | |  | | 46.79 | | 14 | 33.42 | | Right middle frontal gyrus | | 6 |
|  | | |  | | 39.75 | | 36.8 | 12.04 | | Right inferior frontal gyrus | | 46 |
|  | | |  | | 29.97 | | 22.01 | 48.31 | | Right superior frontal gyrus | | 8 |

Peak coordinates and cortical regions in each cluster are ordered downwards from the highest peak Z-score.

All clusters contained ≥100 voxels and were significant at p<0.05 FWE-corrected for multiple comparisons at cluster-level. Abbreviations: BA, Brodmann area; HC, healthy controls; MCI-AD, Mild cognitive impairment due to Alzheimer’s disease; EMCI, Early mild cognitive impairment; LMCI, Late mild cognitive impairment; VOI, volumes of interest; NPTX2, neuronal pentraxin-2; Res_NPTX2, residual NPTX2 component (as defined in the main text).
